# Society for Cardiovascular Magnetic Resonance 2021 cases of SCMR and COVID-19 case collection series

**DOI:** 10.1186/s12968-022-00872-2

**Published:** 2022-07-04

**Authors:** Jason N. Johnson, Daniel B. Loriaux, Elizabeth Jenista, Han W. Kim, Anna Baritussio, Estefania De Garate Iparraguirre, Chiara Bucciarelli-Ducci, Vanessa Denny, Brian O’Connor, Saira Siddiqui, Kana Fujikura, Charles W. Benton, Jonathan W. Weinsaft, Jonathan Kochav, Jiwon Kim, Chaitanya Madamanchi, Michael Steigner, Raymond Kwong, Diego Chango-Azanza, Mónica Chapa, Sandra Rosales-Uvera, Puja Sitwala, Peter Filev, Anurag Sahu, Jason Craft, George J. Punnakudiyil, Viraj Jayam, Farah Shams, Sean G. Hughes, Jonan C. Y. Lee, Edward A. Hulten, Kevin E. Steel, Sylvia S. M. Chen

**Affiliations:** 1 Division of Pediatric Cardiology and Pediatric Radiology, The University of Tennessee Health Science Center, Memphis, TN USA; 2 Division of Cardiology, Duke University Medical Center, Durham, NC USA; 3 Department of Cardiac, Thoracic, Vascular Sciences, and Public Health, University of Padua, Padua, Italy; 4 Bristol Heart Institute, NIHR Bristol Biomedical Research Centre University Hospitals Bristol NHS Foundation Trust and University of Bristol, Bristol, UK; 5 Goryeb Children’s Hospital, Atlantic Health System, Morristown, NJ USA; 6 Robert Wood Johnson University Hospital, New Brunswick, NJ USA; 7 Department of Health and Human Services, National Heart, Lung, and Blood Institute, National Institutes of Health, Bethesda, MD USA; 8 Weill Cornell Medicine, New York, NY USA; 9 Cardiovascular Medicine, University of Michigan, Ann Arbor, MI USA; 10 Brigham and Women’s Hospital, Boston, MA USA; 11 National Institute of Medical Sciences and Nutrition Salvador Zubirán, Mexico City, Mexico; 12 Self Regional Healthcare, Greenwood, SC USA; 13 Emory University Hospital, Atlanta, GA USA; 14 Dematteis Research Center, Greenvale, NY USA; 15 Infectious Diseases, St Francis Hospital, Roslyn, NY USA; 16 Division of Cardiovascular Medicine, Vanderbilt University Medical Center, Nashville, TN USA; 17 Department of Radiology and Imaging, Queen Elizabeth Hospital, Hong Kong, China; 18 Volunteer, Private Consultant, Bethesda, MD USA; 19 PeaceHealth Medical Group, Bellingham, WA USA; 20 Department of Adult Congenital Heart Disease and Cardiology, The Prince Charles Hospital, Brisbane, Australia

## Abstract

The Society for Cardiovascular Magnetic Resonance (SCMR) is an international society focused on the research, education, and clinical application of cardiovascular magnetic resonance (CMR). “Cases of SCMR” is a case series hosted on the SCMR website (https://www.scmr.org) that demonstrates the utility and importance of CMR in the clinical diagnosis and management of cardiovascular disease. The COVID-19 Case Collection highlights the impact of coronavirus disease 2019 (COVID-19) on the heart as demonstrated on CMR. Each case in series consists of the clinical presentation and the role of CMR in diagnosis and guiding clinical management. The cases are all instructive and helpful in the approach to patient management. We present a digital archive of the 2021 Cases of SCMR and the 2020 and 2021 COVID-19 Case Collection series of nine cases as a means of further enhancing the education of those interested in CMR and as a means of more readily identifying these cases using a PubMed or similar literature search engine.

**Supplementary Information:**

The online version contains supplementary material available at 10.1186/s12968-022-00872-2.

## Introduction

A big thank you to our wonderful team of Associate Editors and Reviewers for the Society for Cardiovascular Magnetic Resonance (SCMR) website “Cases of SCMR” series. 'Cases of SCMR' was previously referred to as the ‘Case of the Week’ series. This year, we have the privilege to present also, SCMR’s 2020 and 2021 COVID-19 Case Collection. Coronavirus disease 2019 (COVID-19) has had a significant impact worldwide, and it is important to recognize its impact on the heart. The presentation of the COVID-19 cases differ a little to that of Cases of SCMR. The presentations focus on the main clinical findings and features on cardiovascular magnetic resonance (CMR) as opposed to the more complex Cases of SCMR with a more detailed discussion and perspective sections. As previously in Cases of SCMR, we have an international representation of cases, from the United States and Europe, and pleasingly, a case from Mexico. There was a mixture of adult and pediatric cases for Cases of SCMR, demonstrating the broad utility of CMR in assessing and diagnosing cardiovascular disease. We present unexpected and rare diagnoses by CMR and therefore highlighted important information in guiding clinical management. Cases appropriate for Cases of SCMR are those that demonstrate the role of CMR in context of clinical presentation, diagnosis and management; and the way it complements other imaging modalities [1, 2]. COVID-19 cases submissions are now accepted and published under ‘Cases of SCMR’. Please continue to submit illustrative cases to: https://scmr.org/page/SubmitCase and enjoy the cases!

## Case 1: Eosinophilic myocarditis

### Clinical history

**Fig. 1:**
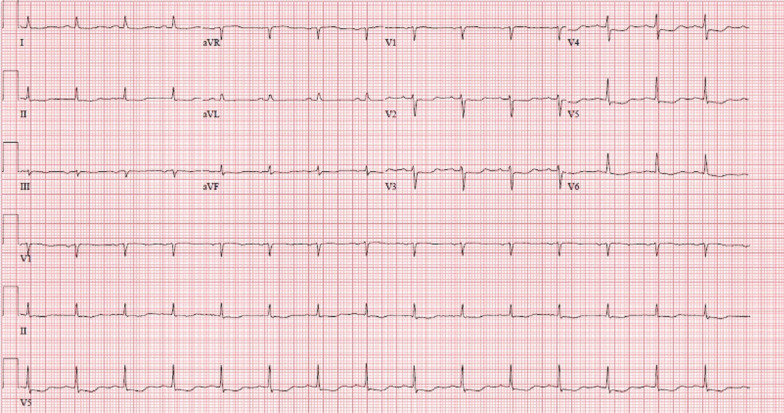
Case 1. Twelve lead electrocardiogram (ECG). Sinus tachycardia with diffuse ST depression

A 70-year-old female with a past medical history of asthma presented to the emergency department for further evaluation of atypical chest pain, orthopnea, and progressive dyspnea on exertion. Initial laboratory findings showed markedly elevated high-sensitivity troponin T (1,200 ng/L), N-terminal-pro-hormone brain natriuretic peptide (NT-pro BNP) (4,400 pg/mL), and hypereosinophilia (6.48 × 10^9/L). Her electrocardiogram (ECG) (Fig. 1 Case 1 Fig. 1) demonstrated sinus tachycardia with diffuse ST depressions, new from prior studies.

**Fig. 2:**
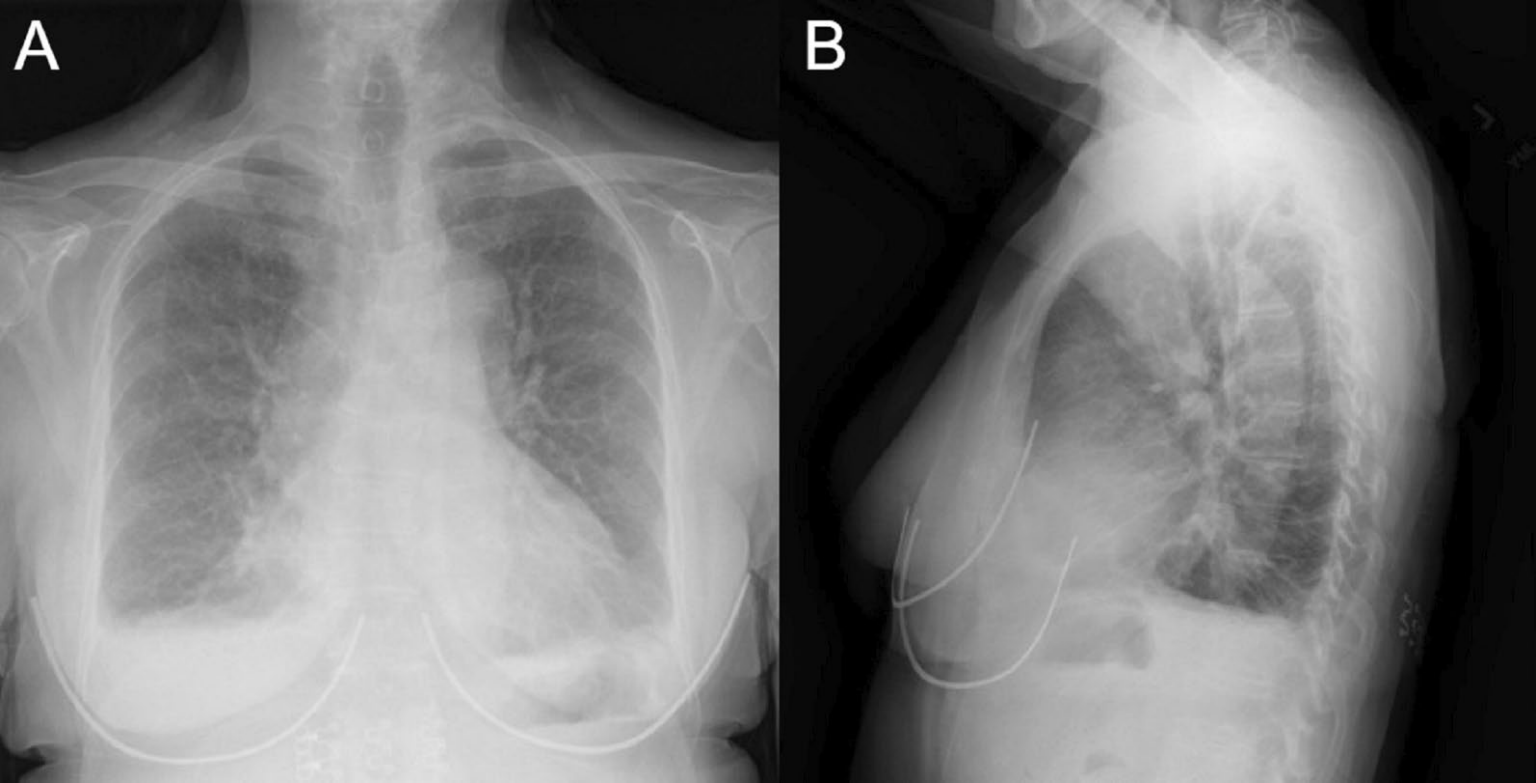
Case 1. Posteroanterior **A** and lateral **B** chest X-ray. Enlarged cardiomediastinal silhouette and small bilateral pleural effusions

A posteroanterior chest X-ray (Fig. 2 Case 1 Fig. 2) was notable for an enlarged cardiomediastinal silhouette with bilateral pleural effusions. A transthoracic echocardiogram (TTE) was notable for normal left ventricular (LV) wall thickness, cavity size, and systolic function. There was a moderate circumferential pericardial effusion and pericardial thickening (Additional file 1: Case 1 Movie 1). A CMR was ordered for further evaluation of suspected myopericarditis.

### CMR findings

**Fig. 3:**
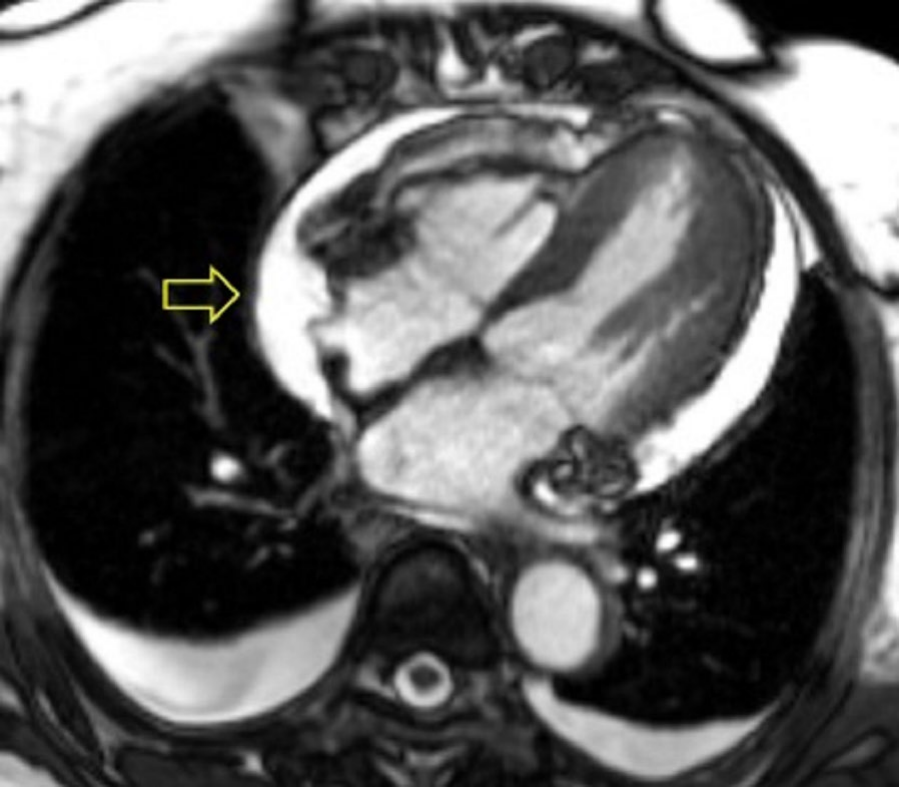
Case 1. Cine balanced steady state free precession (bSSFP) 4 chamber at end-diastole. There is brief right atrial (RA) compression (arrow) present

Cine imaging demonstrated a moderate circumferential pericardial effusion and mild pericardial thickening (Additional file 2: Case 1 Movie 2). LV cavity dimensions were small. Biventricular function was normal. The posterior right atrial (RA) wall briefly collapsed during atrial filling, suggesting increased pericardial pressures (Fig. 3 Case 1 Fig. 3). Importantly, this finding has relatively limited sensitivity (55–60%) and sensitivity (50–68%) for tamponade[3]. The specificity of RA collapse for tamponade improves when the collapse persists for more than 30% of the cardiac cycle, which was not observed in this case.

Native T1 maps were deemed non-diagnostic due to poor co-registration from significant sinus arrhythmia, which led to image acquisition in different portions of the cardiac cycle. Automated motion correction, which adjusts for in-plane motion, may lead to errors in T1 measurements when both in-plane and through plane motion are present due to variations in the amount of partial volume between myocardium and blood. In this circumstance, manual correction leads to the same errors. T2 mapping did not reveal an elevation in T2, although only one slice was imaged.

**Fig. 4:**
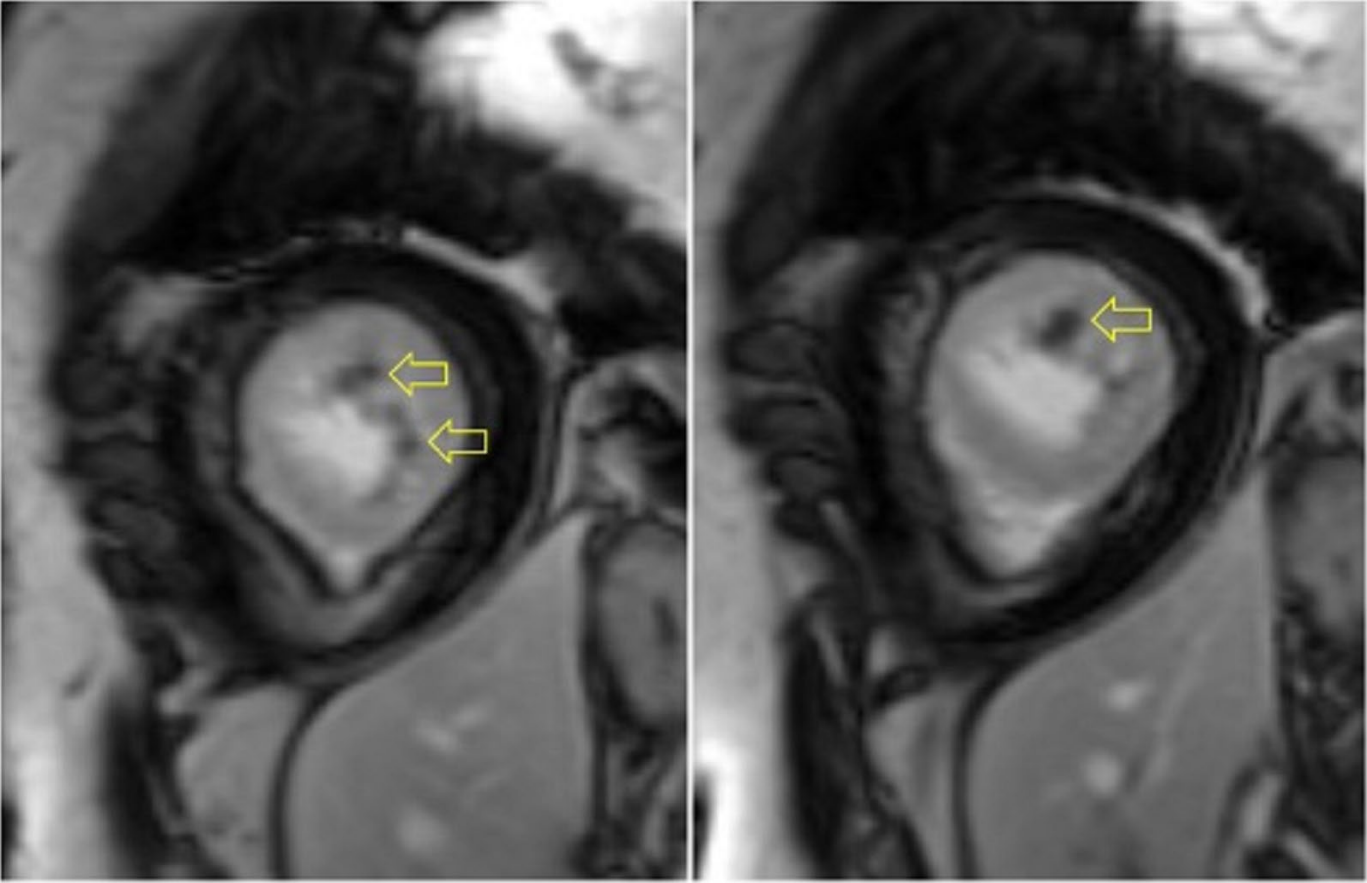
Case 1. Short axis post contrast phase sensitive inversion recovery with prolonged inversion time. There are multiple intracardiac thrombi (arrows)

Following administration of gadolinium contrast, single shot delayed enhancement imaging was performed using an inversion time of 875 ms (the null time for thrombus at 3 T) to screen for intracardiac thrombus. Several hypo-enhanced (dark) regions were present along the endocardial surface of the LV (Fig. 4 Case 1 Fig. 4), suggesting the presence of multiple LV thrombi.

**Fig. 5:**
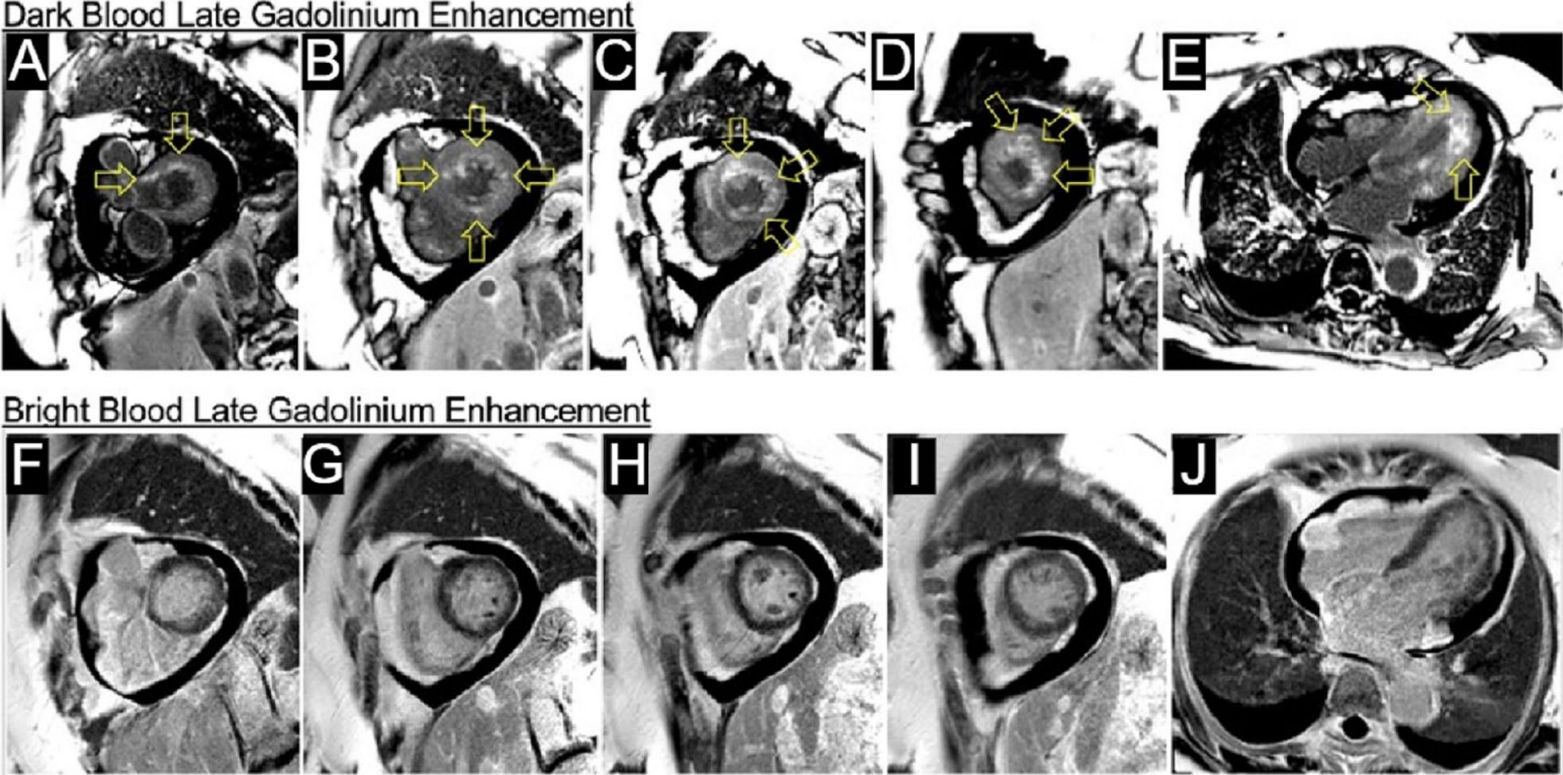
Case 1. Short axis and four chamber dark blood **A**–**E** and bright blood **F**–**J** late gadolinium enhancement (LGE) images. Comparison of bright and dark blood LGE imaging (FIDDLE technique). Dark blood LGE imaging reveals endocardial hyperenhancement not evident on conventional bright blood imaging due to superior contrast between scar and the blood pool

Flow independent dark blood delayed enhancement (FIDDLE) imaging demonstrated regions of patchy, endocardial hyperenhancement involving the distal lateral wall, apex, anterior wall, and papillary muscles. These regions were not well visualized by conventional late gadolinium enhancement (LGE) imaging, likely due to the similar T1 of blood and the involved myocardium (Fig. 5 Case 1 Fig. 5). Pericardial enhancement was also noted.

### Conclusion

**Fig. 6:**
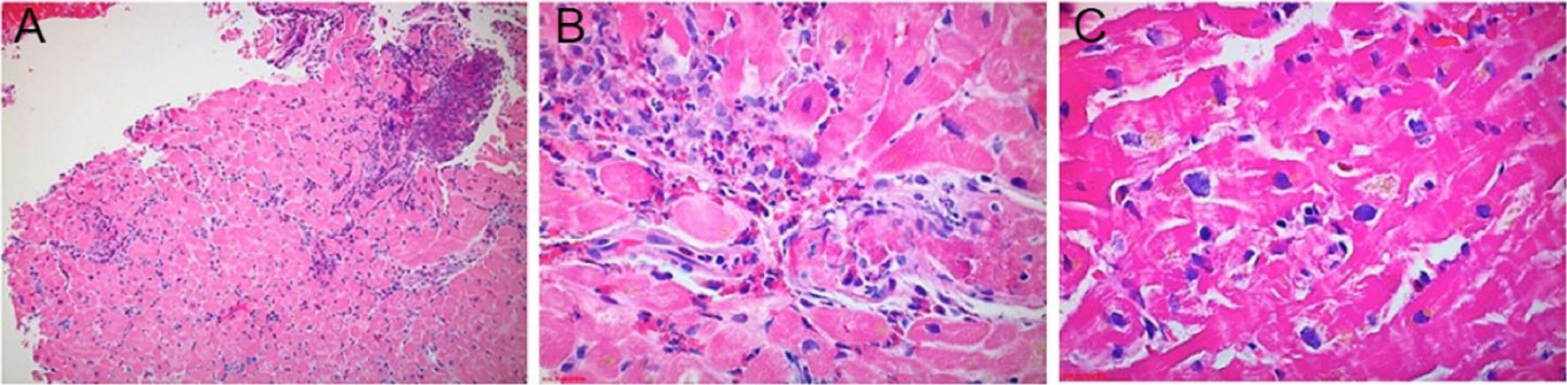
Case 1. Endomyocardial biopsy at 100 power (**A**), 200 power (**B**), and 400 power (**C**). Perivascular and interstitial inflammatory infiltrate with prominent eosinophils

This patient’s CMR findings (pericardial thickening and enhancement, LV thrombi, and patchy endocardial hyperenhancement of the distal segments) in the setting of new heart failure and severe hypereosinophilia were most consistent with acute eosinophilic myocarditis. A confirmatory endomyocardial biopsy was performed (Fig. 6 Case 1 Fig. 6).

The biopsy sample confirmed a moderate perivascular and interstitial inflammatory cell infiltrate with prominent eosinophils. High dose steroid therapy was initiated with an extended prednisone taper. At the patient’s one-month post-discharge follow-up, she reported resolution of her dyspnea, orthopnea, and chest pain. Her laboratory abnormalities, ECG changes, and TTE findings normalized.

### Perspective

Hypereosinophilic syndromes (HES) are a heterogenous group of conditions defined by end-organ damage due to the cytotoxic effects of eosinophilic infiltrates. The diagnosis is rare, with a reported prevalence of 0.036 cases per 100,000[4]. Approximately half of all HES cases include cardiac involvement manifesting as eosinophilic myocarditis[5, 6]. Eosinophilic myocarditis progresses across three clinical stages: an acute necrotic stage, intermediate thromboembolic stage, and final fibrotic stage. This discussion will highlight the clinical features of each stage as well as the corresponding CMR findings.

The acute necrotic stage of eosinophilic myocarditis, which is usually clinically silent, is characterized by markedly elevated troponin and NT-pro BNP levels in the setting of absent coronary artery disease and normal ventricular function. Peripheral eosinophilia will remain absent in 25% of eosinophilic myocarditis cases and cannot be used as a reliable indicator. CMR is highly sensitive for even the earliest stage of eosinophilic myocarditis, characteristically showing hyperintensity on T1 or T2 weighted imaging with subendocardial late gadolinium enhancement (LGE)[6–8].

Transition to the thromboembolic stage of eosinophilic myocarditis is variable in timing and may occur 1 day to 3 months after the acute necrotic stage[9]. Symptom onset most often occurs during this second stage and might include fever (25–30% of cases), dyspnea (40–70%), atypical chest pain (40–45%), and thromboembolic complications (5%)[4, 10–12]. Intracavitary thrombogenesis in eosinophilic myocarditis is driven by eosinophil peroxidase mediated production of hypothiocyanous acid, which enhances tissue factor expression and clot deposition in regions of dense eosinophilic infiltration[13]. This mechanism permits subendocardial thrombi to form despite the absence of wall motion abnormalities. Clot formation is easily visualized on CMR during this second stage through selective nulling of avascular tissue using prolonged inversion times[14].

The final fibrotic stage of eosinophilic myocarditis is defined by scarring of the damaged myocardium, a restrictive cardiomyopathy, and new onset heart failure[15]. Preferential accumulation of thrombofibrotic material between the ventricular aspect of the posterior mitral leaflet and the mural endocardium of the LV free wall may present as new mitral regurgitation[16]. Superimposed on the findings of the first and second stages, the presence of dilated atria (due to restrictive physiology and diastolic dysfunction) is a CMR finding that can be used to identify the fibrotic stage[17].

Eosinophilic myocarditis is a challenging diagnosis that demands a high clinical index of suspicion and a low threshold for acquisition of CMR. In our case, the diagnosis hinged upon the combination of localized hypertrophy evident on cine imaging and endocardial hyperenhancement on FIDDLE[18]. To our knowledge, this is the first case to highlight the use of FIDDLE in the diagnosis of eosinophilic myocarditis and demonstrates the importance of separating blood signal from hyperenhanced myocardium in patients with this disease.

At our center, we have used FIDDLE for clinical purposes since 2010. We have found that that utility of dark blood delayed enhancement extends beyond the detection of myocardial infarction (MI) and improves the visualization of hyperenhanced myocardium in a variety of patients with non-ischemic cardiomyopathies. Currently, FIDDLE is not commercially available; however, the works in progress sequence can be requested by participating members (with software compatibility) of the SCMR Registry.

## Case 2: Left ventricular hypertrophy: the other side of the coin

### Clinical history

A 34-year-old naval officer, otherwise fit and healthy, with no known family history of cardiomyopathies, presented with chest pain, poorly tolerated atrial fibrillation and progressive hypotension. A TTE showed asymmetric septal hypertrophy, with preserved LV ejection fraction (LVEF) and severe left atrial (LA) enlargement. A high sensitive troponin I was elevated, with persistent chest pain, a coronary angiogram was performed showing unobstructed coronaries and a chest computed tomography (CT) showed no evidence for pulmonary embolism. Blood tests showed mildly increased inflammatory markers. A CMR was requested.

### CMR findings

**Fig. 7:**
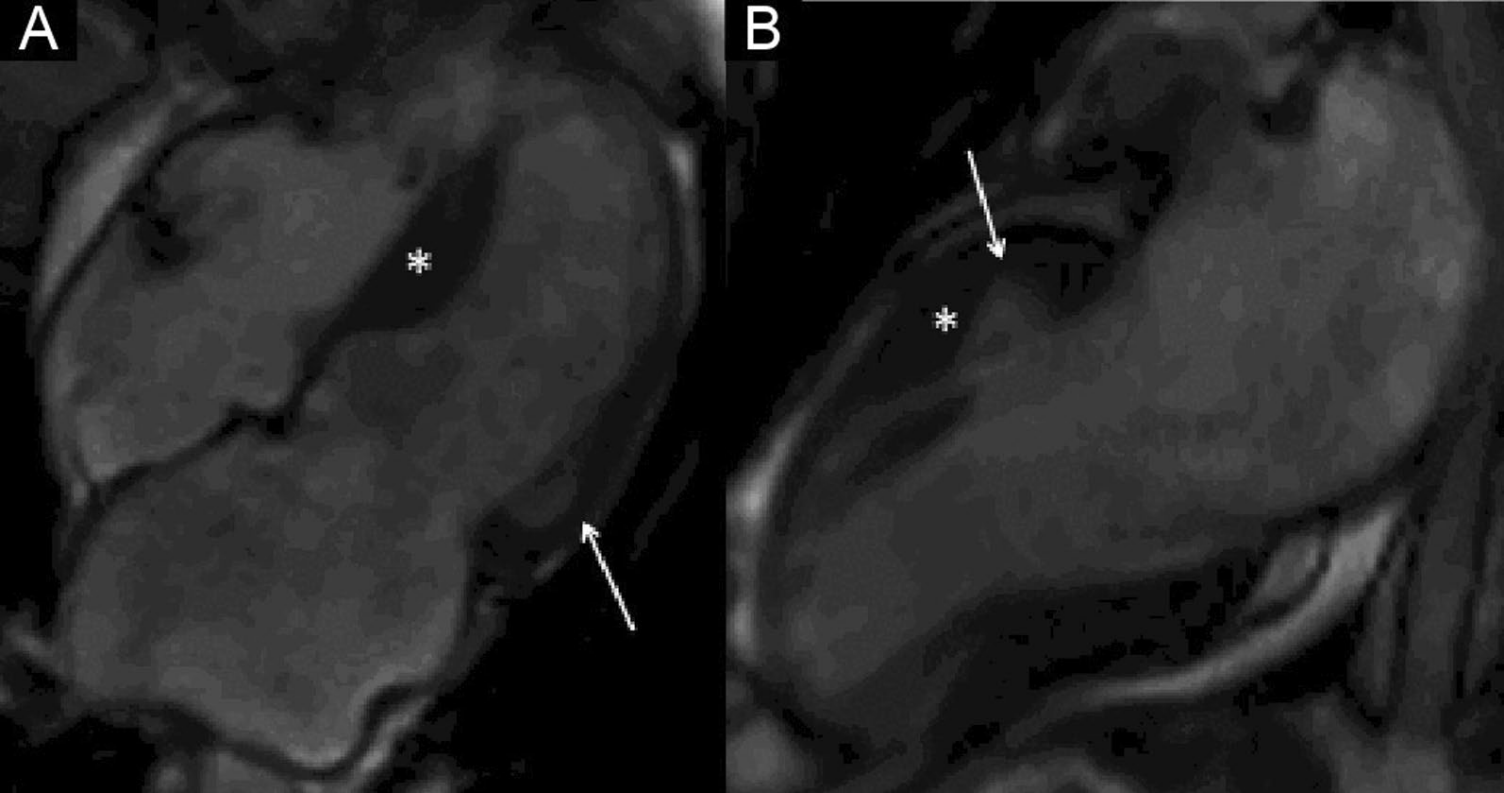
Case 2. Figure 1. Four **A** and two chamber **B** bSSFP cine sequences. There are septal and anterior left ventricular (LV) hypertrophy (LVH) (white asterisk) and myocardial crypts of the basal anterior and anterolateral walls (white arrow) present

The LV had normal volumes (LV end-diastolic volume indexed (LVEDVI) 86 ml/m^2^, LV end-systolic volume indexed (LVESVI) 38 ml/m^2^) with low normal LVEF (56%). There was a mixed pattern of LV hypertrophy (LVH) and thinning, with asymmetric hypertrophy in portions of the anterior and septal segments (Fig. 7 Case 2 Fig. 1, white asterisk), from base to mid-cavity (max 15 mm basal), and wall thinning of the basal and mid-cavity lateral wall, extending to the apical segments (min 4 mm apical anterior wall); hypertrophied segments appeared stiff, while the thinned segments were mildly hypokinetic. Two large myocardial crypts were noted in the basal anterior and anterolateral walls (Fig. 7 Case 2 Fig. 1, white arrows; Additional file 3: Case 2 Movie 1).

**Fig. 8:**
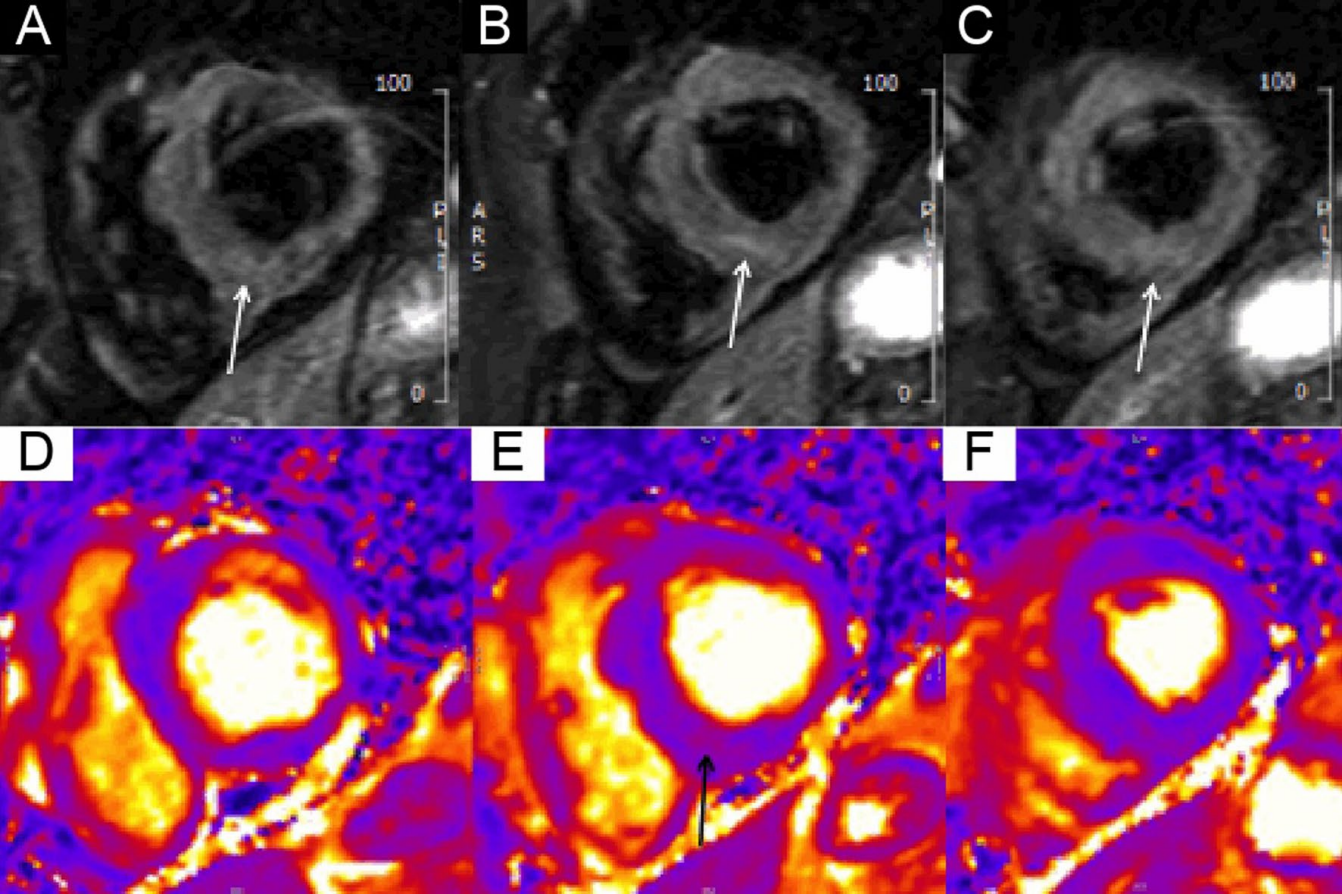
Case 2. Figure 2. Basal short axis T2-weighted sequences (**A**–**C**). Basal short axis T2-mapping images (**D**–**F**). The T2-weighted images show mid-wall increased signal intensity in the basal inferoseptum, consistent with myocardial oedema (white arrows). The T2-mapping images show increased T2 values in the basal inferoseptum (black arrow)

The right ventricle (RV) had normal wall thickness, volumes (RV end-diastolic volume indexed (RVEDVI) 74 ml/m^2^, RV end-systolic volume indexed (RVESVI) 24 ml/m^2^) and systolic function (RV ejection fraction, RVEF 65%). The LA was severely dilated (50cm^2^). On T2 short tau inversion recovery (STIR) imaging, there was mid-wall myocardial oedema of the basal inferoseptum (Fig. 8 Case 2 Fig. 2, A–C, white arrows), which corresponded to an area of higher T2 values on T2 mapping (Fig. 8 Case 2 Fig. 2, E, black arrow).

**Fig. 9:**
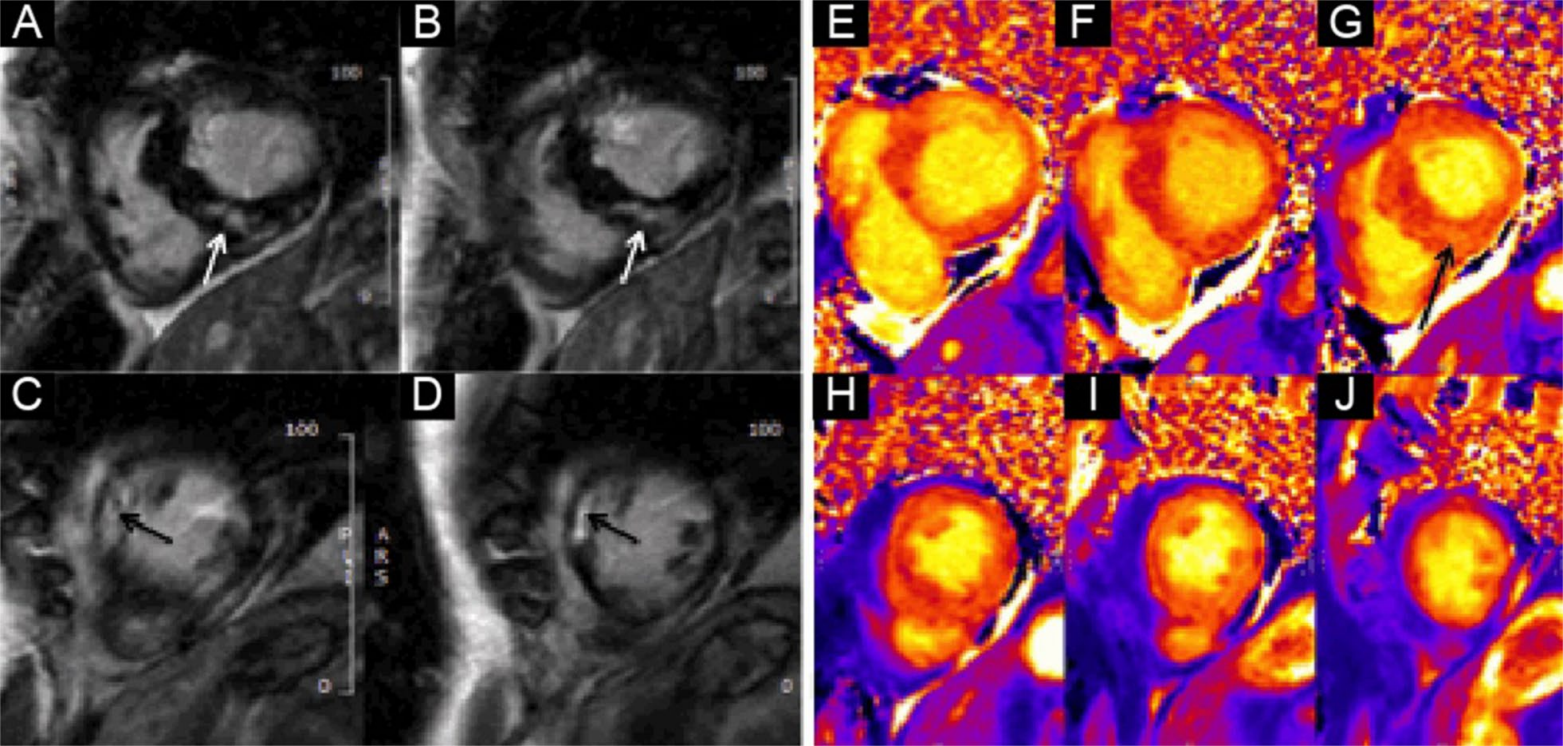
Case 2. Figure 3. Basal to mid-wall short axis phase sensitive inversion recovery (PSIR) post-contrast sequences (**A**–**D**). Short axis native T1 mapping images (**E**–**J**). The post-contrast images show patchy mid-wall myocardial LGE of the inferoseptum (white arrows) and in the mid-cavity anterior wall (black arrows). The native T1 mapping images show increased native T1 values in the basal inferoseptum (black arrow)

On post-contrast images, patchy mid-wall myocardial LGE was noted in the inferoseptum (Fig. 9 Case 2 Fig. 3, A, B, white arrows), from base to apex, and in the mid-cavity and apical anterior wall (Fig. 9 Case 2 Fig. 3, C, D, black arrows). On native T1 mapping, increased T1 values were noted, especially in the hypertrophied inferoseptum (Fig. 9 Case 2 Fig. 3, G, black arrow). Mediastinal lymphadenopathy was also noted.

### Conclusion

CMR findings were consistent with an underlying cardiomyopathic process. The presence of asymmetric septal and anterior hypertrophy with non-ischemic fibrosis, could lead to a diagnosis of hypertrophic cardiomyopathy (HCM) with replacement fibrosis, but cardiac sarcoid, especially with evidence of septal oedema and mediastinal lymphadenopathy, could also be possible. An infective process could also be possible, given the presence of reactive oedema in the hypertrophied segments, mediastinal lymphadenopathy and increased inflammatory markers, although it is usually less likely related to LVH.

**Fig. 10:**
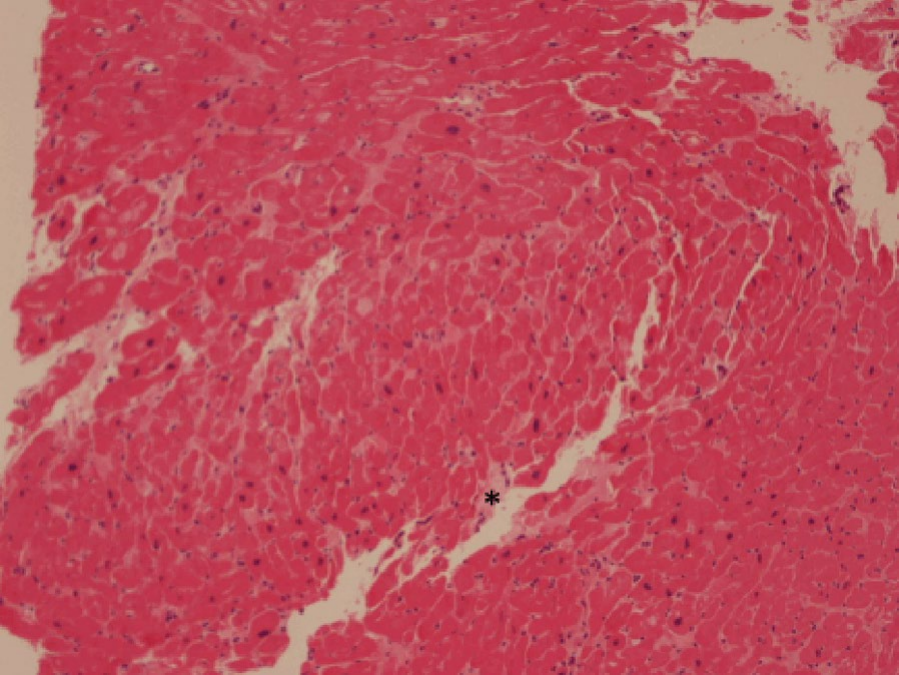
Case 2. Figure 4. Endomyocardial biopsy. Hypertrophic myocytes with interstitial fibrosis (black asterisk) are present

However, there was an unusual pattern of LVH and wall thinning, which is frequently described in mitochondrial cardiomyopathies. Barth’s syndrome for example, which is secondary to an X-linked related mitochondrial dysfunction, is sometimes characterized by transition between dilated and hypertrophic cardiac morphology[19]. A suspicion of mitochondrial cardiomyopathy was raised, and given persistence of chest pain with elevated high sensitive troponin, an endomyocardial biopsy (EMB) was performed, showing hypertrophied myocytes with focal interstitial fibrosis (Fig. 10 Case 2 Fig. 4, black asterisk), not consistent with HCM, sarcoidosis or amyloidosis, and with an increased number of mitochondria within the myocytes on electron microscopy, the latter being frequently reported in mitochondrial cardiomyopathies.

### Perspective

Myocardial LVH is the final expression of different cardiomyopathies, with widely different prognosis and requiring different management strategies. CMR has a pivotal role in differential diagnosis of hypertrophic phenotypes due to its unique tissue characterization properties [20]; it is a powerful diagnostic tool, which should however never be separated from patients’ clinical history, laboratory tests, physical examination and other imaging findings.

The unusual pattern of LVH and wall thinning raised the suspicion of mitochondrial cardiomyopathy, leading to EMB that excluded HCM and sarcoid, while showing an increased number of mitochondria, which is frequently described in mitochondrial cardiomyopathies. Although rare, mitochondrial cardiomyopathies are characterized by a wide range of cardiac abnormalities, ranging from conduction abnormalities, to hypertrophic or dilated phenotypes, with variable LGE patterns, such as mid-wall basal inferolateral wall or diffusely distributed LGE. CMR aids in the diagnosis of mitochondrial cardiomyopathies, with relevant implications on patients’ management given the increased mortality risk associated with this disease [21].

Based on EMB and CMR findings, a diagnosis of mitochondrial cardiomyopathy seems likely and should prompt wider genetic testing as further diagnostic work-up.

The CMR of Case 2 (Additional file CMR Link, https://www.cloudcmr.com/5457-1973-7918-0194/).

## Case 3: A case of arrhythmogenic cardiomyopathy with left ventricular involvement

### Clinical history

**Fig. 11:**
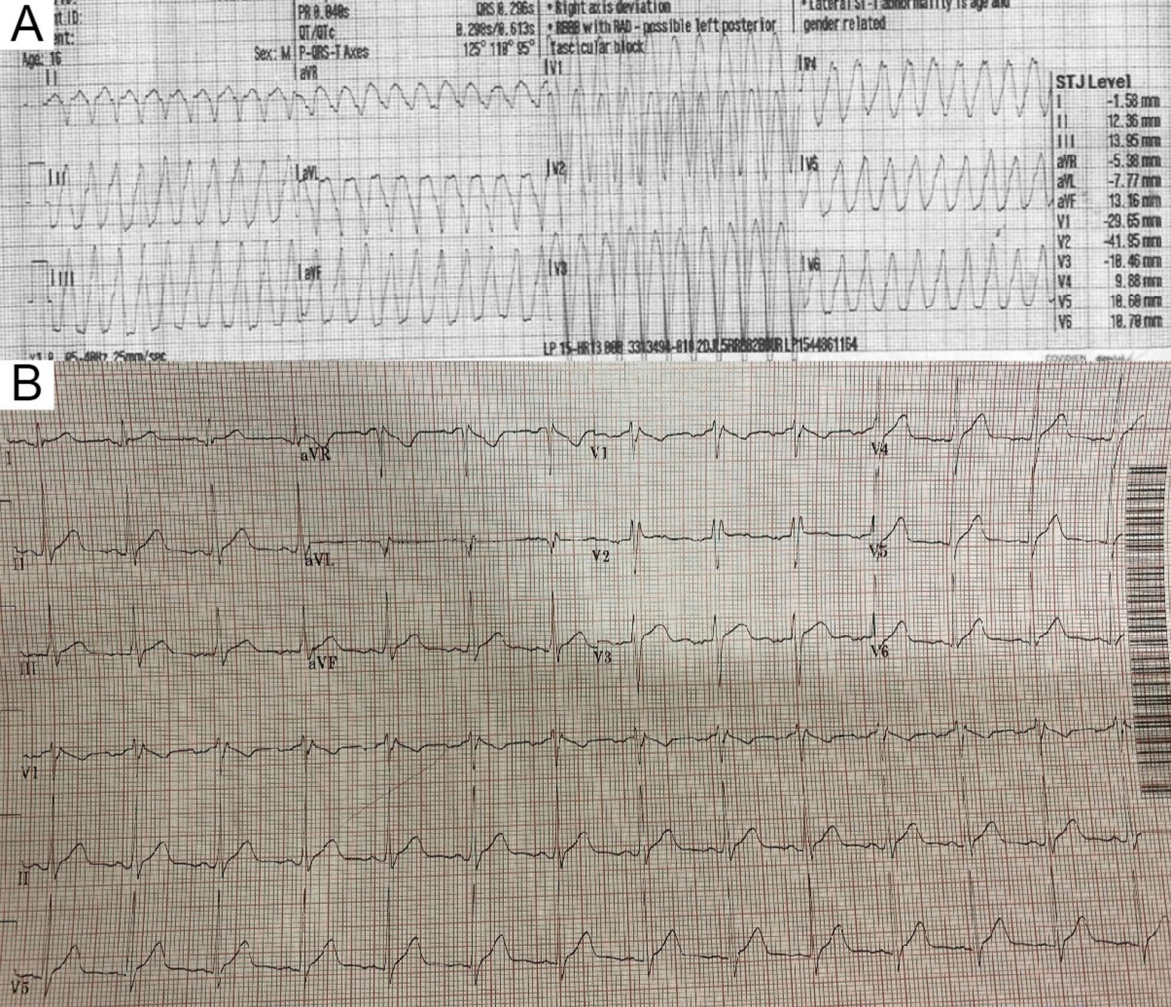
Case 3. Figure 1. 12 lead ECG demonstrating non-sustained ventricular tachycardia originating from the right ventricular outflow tract (**A**). Baseline ECG (**B**)

A 16-year-old highly athletic boy with no significant past medical history or family history, presented with recurrent presyncope. During a basketball game, he developed an hour-long episode of dizziness, pallor, and diaphoresis. Emergency medical services obtained a 12-lead ECG and found him to be in monomorphic ventricular tachycardia (VT) at a rate of 250 beats per minute (bpm), and successfully cardioverted him. He was admitted to an outside hospital where a limited TTE reportedly demonstrated normal cardiac anatomy and function (Additional file 4: Case 3 Movie 1). An ECG obtained during a brief episode of non-sustained VT demonstrated origin from the RV outflow tract (Fig. 11 Case 3 Fig. 1, A). His baseline ECG was normal with an RSR’ pattern in V1 and V2 (Fig. 11 Case 3 Fig. 1, B). He was referred for CMR for further evaluation.

### CMR findings

CMR with balanced steady state free precession (bSSFP) cine imaging, black blood T1-weighted imaging with and without fat saturation, T1 parametric mapping, and LGE imaging was performed.

**Fig. 12:**
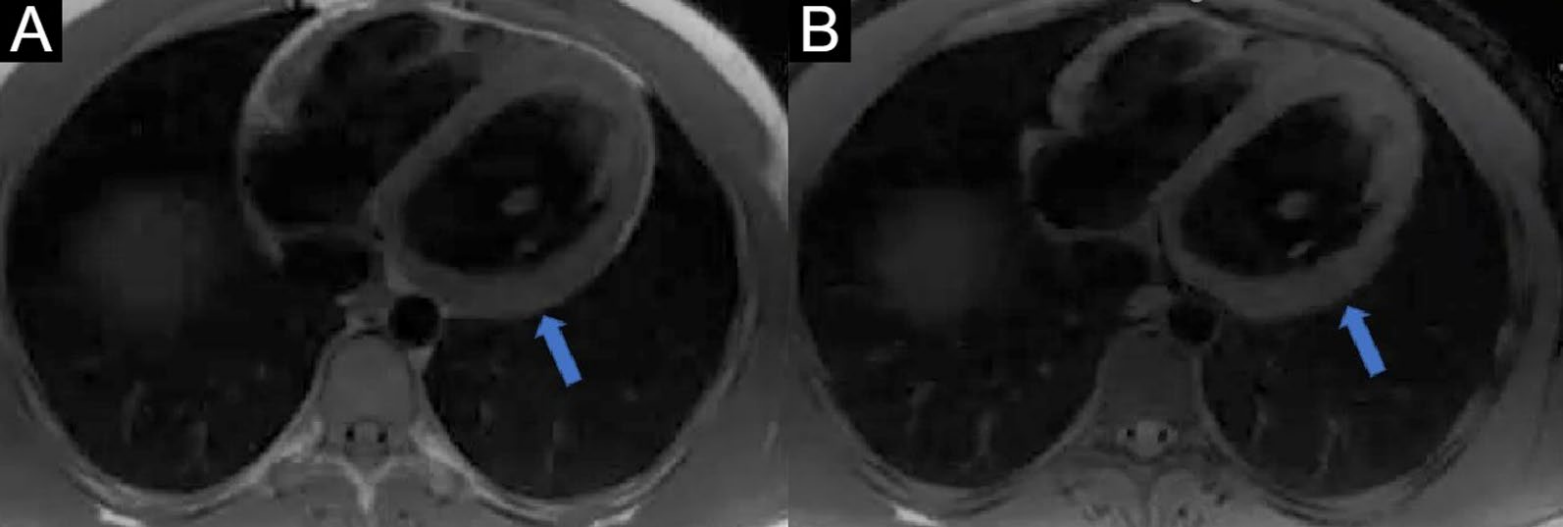
Case 3. Figure 2. Black blood T1-weighted imaging without **A** and with **B** fat saturation. Possible epicardial fat infiltration of the lateral LV (arrows)

**Fig. 13:**
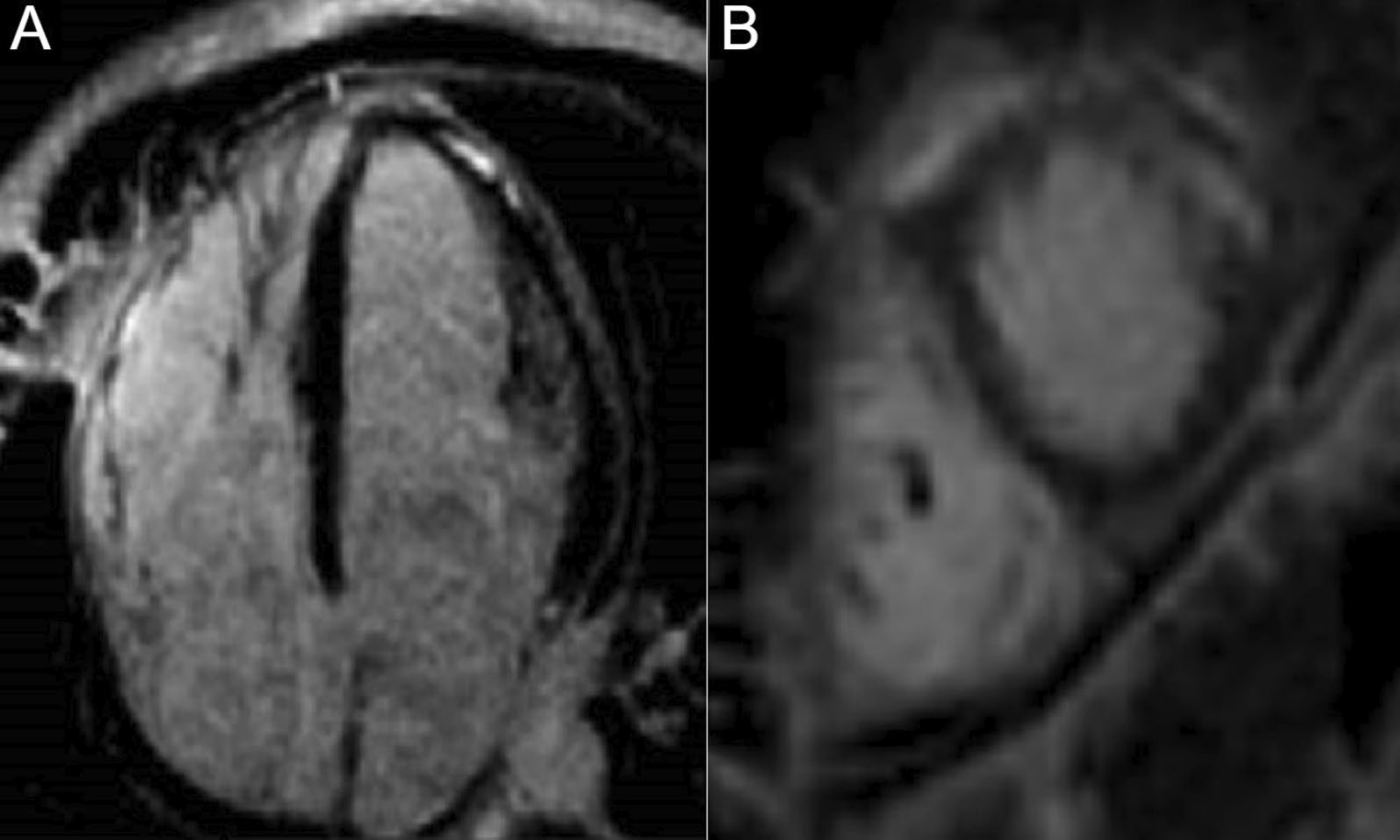
Case 3. Figure 3. LGE on 4-chamber **A** and short axis **B** views. Epicardial to mid-myocardial LGE of the LV apical lateral wall

4-chamber bSSFP cine images demonstrated RV dilation with RV dyskinesia (Additional file 5: Case 3 Movie 2). The LV lateral wall appeared irregular (Additional file 5: Case 3 Movie 2). 2-chamber bSSFP RV cine demonstrated RV microaneurysms along the lateral free wall, consistent with the “accordion sign” (Additional file 6: Case 3 Movie 3, A). 2-chamber bSSFP LV cine demonstrated normal LV systolic function (Additional file 6: Case 3 Movie 3, B). The short axis cine demonstrated a dilated RV (RVEDVI 155 ml/m^2^) with globally mildly reduced systolic function, with a mildly depressed RVEF of 42%. There was normal LV systolic function with an LVEF of 55% with no regional dysfunction (Additional file 7: Case 3 Movie 4). The LV was mildly dilated with an LVEDVI of 122 ml/m^2^ (Additional file 7: Case 3 Movie 4). T1-weighted fast-spin echo black blood imaging with and without fat saturation suggested possible epicardial fat infiltration of the lateral LV (Fig. 12 Case 3 Fig. 2). Parametric mapping with T1 mapping demonstrating normal mean native T1 signal. On LGE imaging, there was subepicardial to mid-wall enhancement at the LV apical lateral segment (Fig. 13 Case 3 Fig. 3).

### Conclusion

CMR findings fulfilled the major criteria for arrhythmogenic right ventricular cardiomyopathy (ARVC) developed by the 2010 ARVC Task Force[22]. Recent case studies have demonstrated biventricular disease involvement is more common than previously reported, prompting the term ARVC to be relabeled as arrhythmogenic cardiomyopathy [23]. His initial ECG showed monomorphic VT suspicious for arrhythmogenic cardiomyopathy. To determine whether he required an implantable cardioverter defibrillator (ICD) alone vs. ICD placement and VT ablation, he underwent an isoproterenol challenge test, which was positive. Subsequently, genetic testing found him to be positive for the desmoglein-2 (DSG2) missense mutation. CMR demonstrated focal RV dyskinesia with “accordion sign” and the RVEDVI measured greater than > 110 mL/m^2^, with a RVEF between 40 to 45%. He thereby satisfied two major diagnostic criteria for arrhythmogenic cardiomyopathy with positive genetic testing and RV findings on CMR. In addition, the presence of subepicardial to mid-wall LGE at the LV apex and possible LV fibrofatty infiltration on T1-weighted imaging was consistent with arrhythmogenic cardiomyopathy with LV involvement. He ultimately had an ICD placed and nadolol was initiated.

### Perspective

Arrhythmogenic cardiomyopathy is a rare inherited disorder that causes fibrofatty replacement of cardiac tissue[24, 25]. This is a known cause of cardiac death in young athletes, typically men less than 35 years old[24]. Presenting symptoms are generally non-specific and may include palpitations, fatigue, syncope, or even aborted sudden cardiac death. CMR is a valuable diagnostic tool that can identify arrhythmogenic cardiomyopathy with and without LV involvement and guide clinical management. CMR diagnostic criteria were first established in 1994 and further revised in 2010 [22]. Initial subtle structural changes to the RV can progress to involve both ventricles[22]. Tissue characterization is a vital tool in diagnosing arrhythmogenic cardiomyopathy, particularly in assessment of LV involvement, which may not be apparent on TTE. Studies have shown that subepicardial LV fatty infiltration, similar to our case, is a common finding in arrhythmogenic cardiomyopathy[26, 27]. With the autosomal dominant inheritance pattern, family history is a critical component to this diagnosis. Several genetic variations in the desmosomes have been found, such as the DSG2 seen in our patient. Some genetic variants are associated with an increased risk of malignant arrhythmias. While arrhythmogenic cardiomyopathy guidelines are evolving, the 2019 Heart Rhythm Society consensus statement has incorporated genetic data into the risk stratification for ICD implantation[28].

This case exemplifies the importance of obtaining CMR for suspected arrhythmogenic cardiomyopathy, even in the presence of normal TTE. CMR imaging allowed for more detailed visualization of the ventricles and tissue characterization with parametric mapping and LGE. CMR findings may prompt genetic workup, which in turn could inform prognosis and management for current and future family members.

The CMR of Case 3 (Additional file CMR Link, https://www.cloudcmr.com/8257-1973-8518-0183/).

## Case 4: Newly developed heart failure: what is the etiology?

### Clinical history

70 year-old female nursing home resident with past medical history of hypertension, hypothyroidism, peripheral vascular disease, and obsessive–compulsive disorder presented to emergency department with shortness of breath of supposedly several days duration. She denied chest pain, palpitation, or cough.

Upon arrival to the emergency department, she was in respiratory distress. Her blood pressure was 102/66 mmHg, heart rate was 108 bpm, pO_2_ saturation 88% on room air. She was afebrile and frail (weight 40 kg, body mass index (BMI) 17 kg/m^2^). She was tachypneic with accessory respiratory muscle use. Rhythm was regular, 2/6 systolic murmur was noted at the base. She had + 2 bilateral ankle edema. Chest X-ray showed bilateral diffuse infiltrates. She was placed on bi-level positive airway pressure (BiPAP) with 100% FiO2, and her respiratory status improved with O_2_ saturation of 96%.

**Fig. 14:**
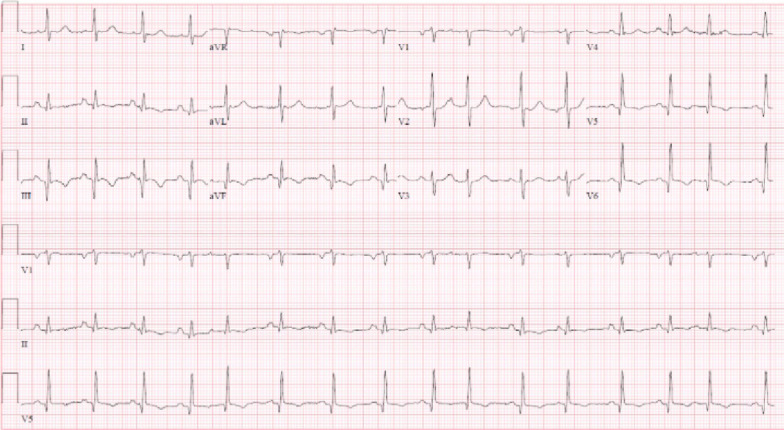
Case 4. Figure 1. ECG on admission. Sinus tachycardia with old inferior myocardial infarction and non-specific T-wave abnormalities in the inferior and lateral leads

Laboratory examination showed mild anemia with hemoglobin 10 g/dL (normal > 12 g/dL), creatinine 1.4 mg/dL (normal < 1.2 mg/dL), blood urea nitrogen (BUN) 47 mg/dL (normal < 30 mg/dL) [3 years prior creatinine was 0.9 mg/dL and BUN 29 mg/dL], estimated glomerular filtration rate (eGFR) 38 mL/min/1.73m^2^ (3-years prior eGFR > 60 mL/min/1.73m^2^), elevated troponin T 2.77 ng/mL (normal < 0.01 ng/mL), and severely elevated NT-pro BNP > 70,000 pg/mL (normal < 125 pg/mL). Her COVID-19 polymerase chain reaction (PCR) test was negative. ECG showed sinus tachycardia with old inferior MI and non-specific T-wave changes in inferior and lateral leads (Fig. 14 Case 4 Fig. 1). The diagnosis of non-ST elevation MI was entertained along with heart failure (HF).

**Fig. 15:**
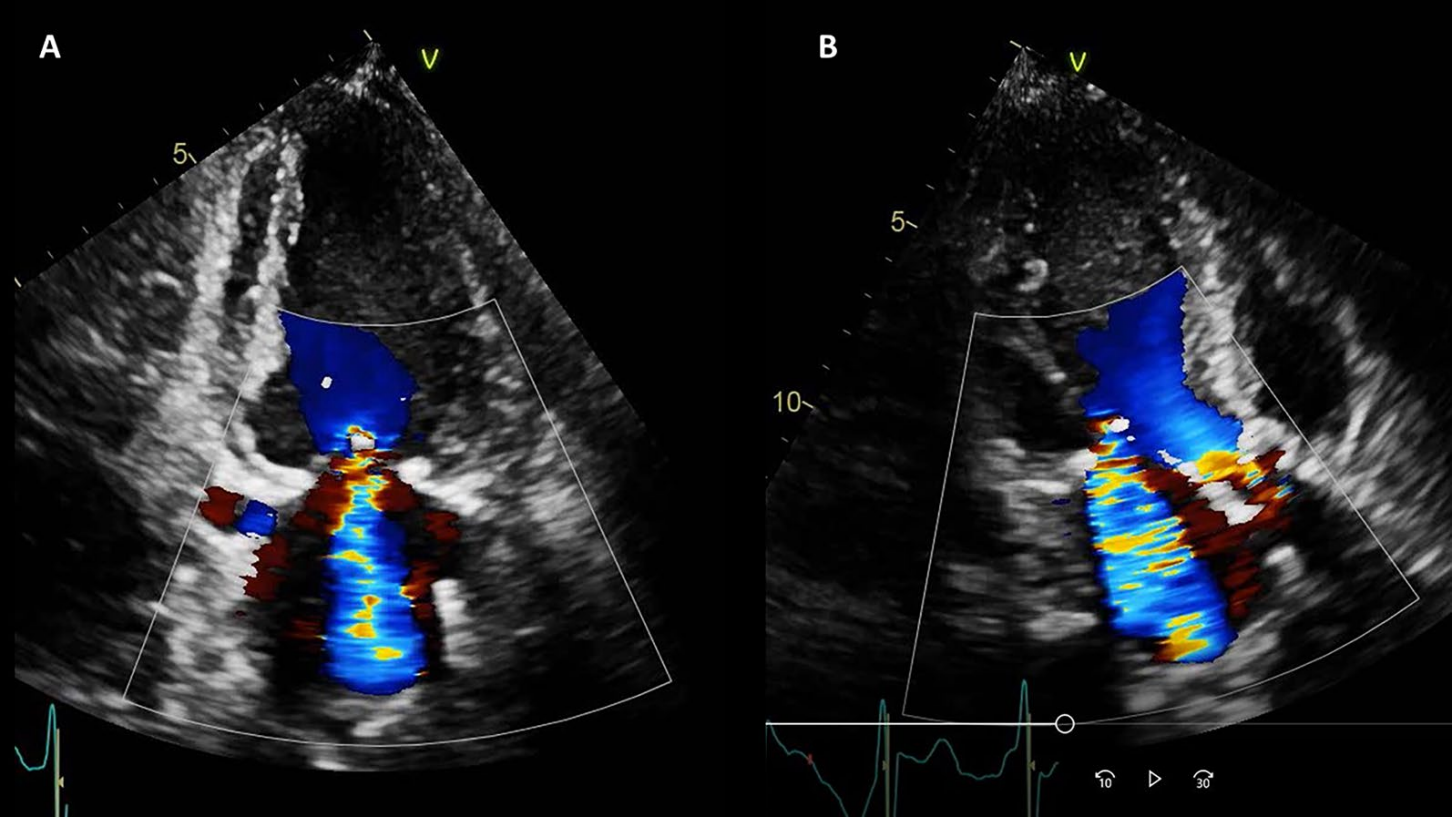
Case 4. Figure 2. Color Doppler echocardiography of **A** apical 2 chamber and **B** apical 3 chamber views. Severe mitral regurgitation and turbulent flow at the aortic valve

On TTE, the LV size was normal and systolic function was moderately decreased, LVEF 35% with significant regional wall motion abnormalities consistent with inferior MI. The mitral valve leaflets were bilaterally tethered, resulting in severe mitral regurgitation (Fig. 15 Case 4 Fig. 2). The aortic valve was calcified with stenosis and regurgitation. The mean pressure gradient across the aortic valve was 21 mmHg, with maximum aortic valve velocity 3.3 m/s and calculated aortic valve area of 0.9 cm^2^ (aortic valve area index 0.7 cm^2^/m^2^) suggestive of at least moderate aortic stenosis (AS). She had pulmonary artery hypertension with estimated RV systolic pressure of 60 mmHg. An invasive cardiac evaluation was recommended but the patient opted for a conservative strategy with medical management. Upon her request, palliative care team was consulted and she elected to have a do not resuscitate (DNR) / do not intubate (DNI) status. She was medically managed and discharged home after 14 days of hospitalization with aspirin, clopidogrel, metoprolol succinate, atorvastatin, and furosemide. Angiotensin converting enzyme inhibitor was held for hypotension.

**Fig. 16:**
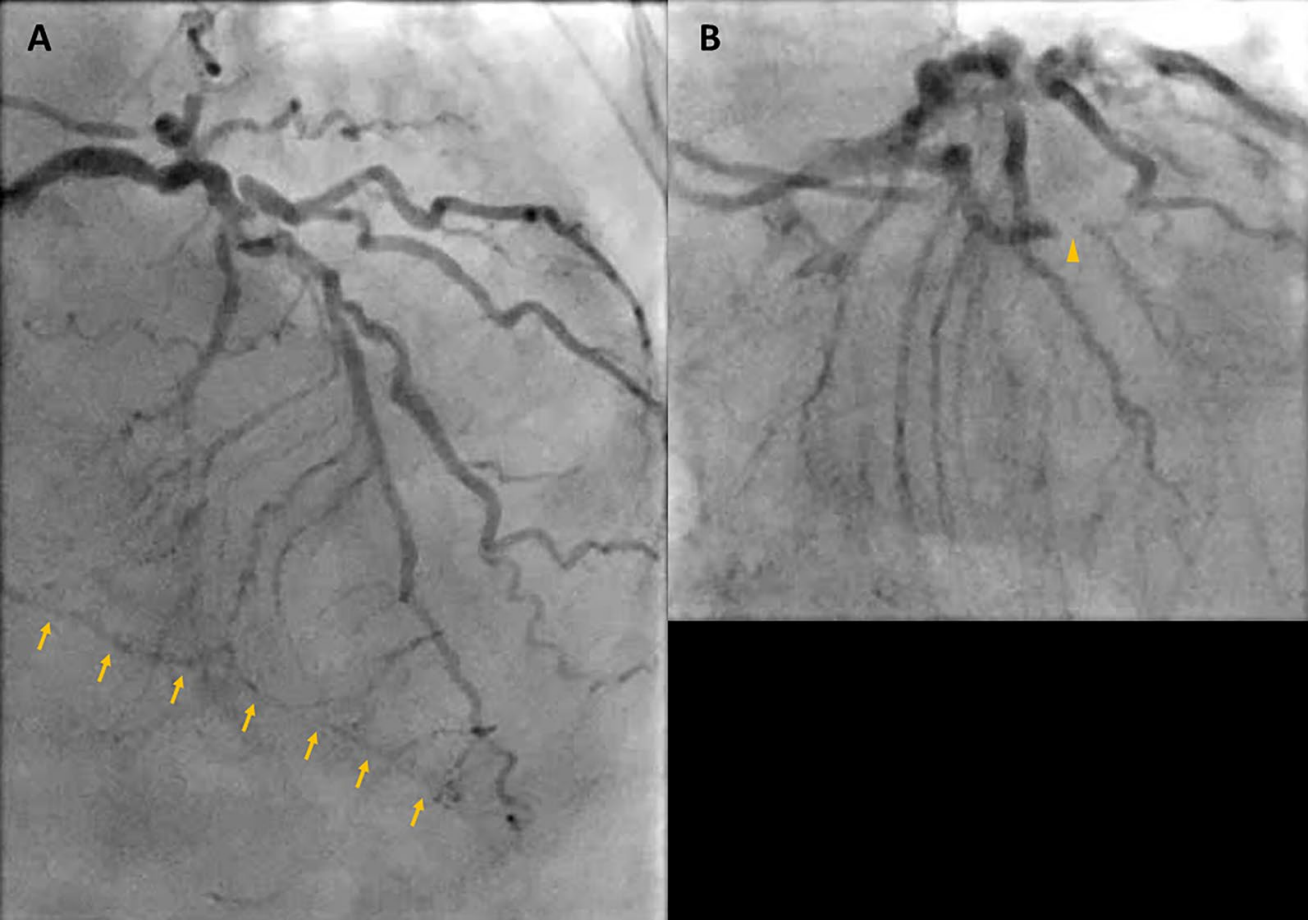
Case 4. Figure 3. Invasive coronary angiography in anterior–posterior cranial view **A** and let anterior oblique caudal view (**B**). Severe multi-vessel coronary disease was present. There was retrograde filling of the right posterior descending artery through contralateral collaterals (yellow arrow) indicating chronic occlusion of right coronary artery (RCA). The left circumflex coronary artery (LCx) is a small caliber vessel with 99% thrombotic subtotal occlusion in its mid segment (yellow arrowhead)

As her shortness of breath worsened, she returned to the emergency room 9 days after her initial discharge requesting full treatment options including invasive procedures. Invasive coronary angiography showed severe multi-vessel coronary disease with chronic occlusion of the dominant right coronary artery (RCA) filling retrograde through contralateral collaterals, subtotal thrombotic occlusion of a small caliber left circumflex (LCx) artery with thrombolysis in myocardial infarction (TIMI) 1–2 flow, and probable significant disease in the proximal left anterior descending (LAD) artery and proximal large diagonal (Fig. 16 Case 4 Fig. 3). LV end-diastolic pressure was 22 mm Hg. By pullback method, mean gradient across aortic valve was 26 mmHg with a peak–to–peak gradient of 22 mmHg.

Ischemic cardiomyopathy was considered as the major contributor for her severe mitral regurgitation and recurrent HF. Cardiothoracic surgery team was consulted for possible coronary artery bypass grafting (CABG), aortic valve replacement (AVR) and mitral valve repair/replacement. The surgical team was of the opinion that she was a high surgical risk, being especially concerned about her frailty. CMR was ordered to assess myocardial viability as part of her risk stratification (and to plan for the surgery).

### CMR findings

**Fig. 17:**
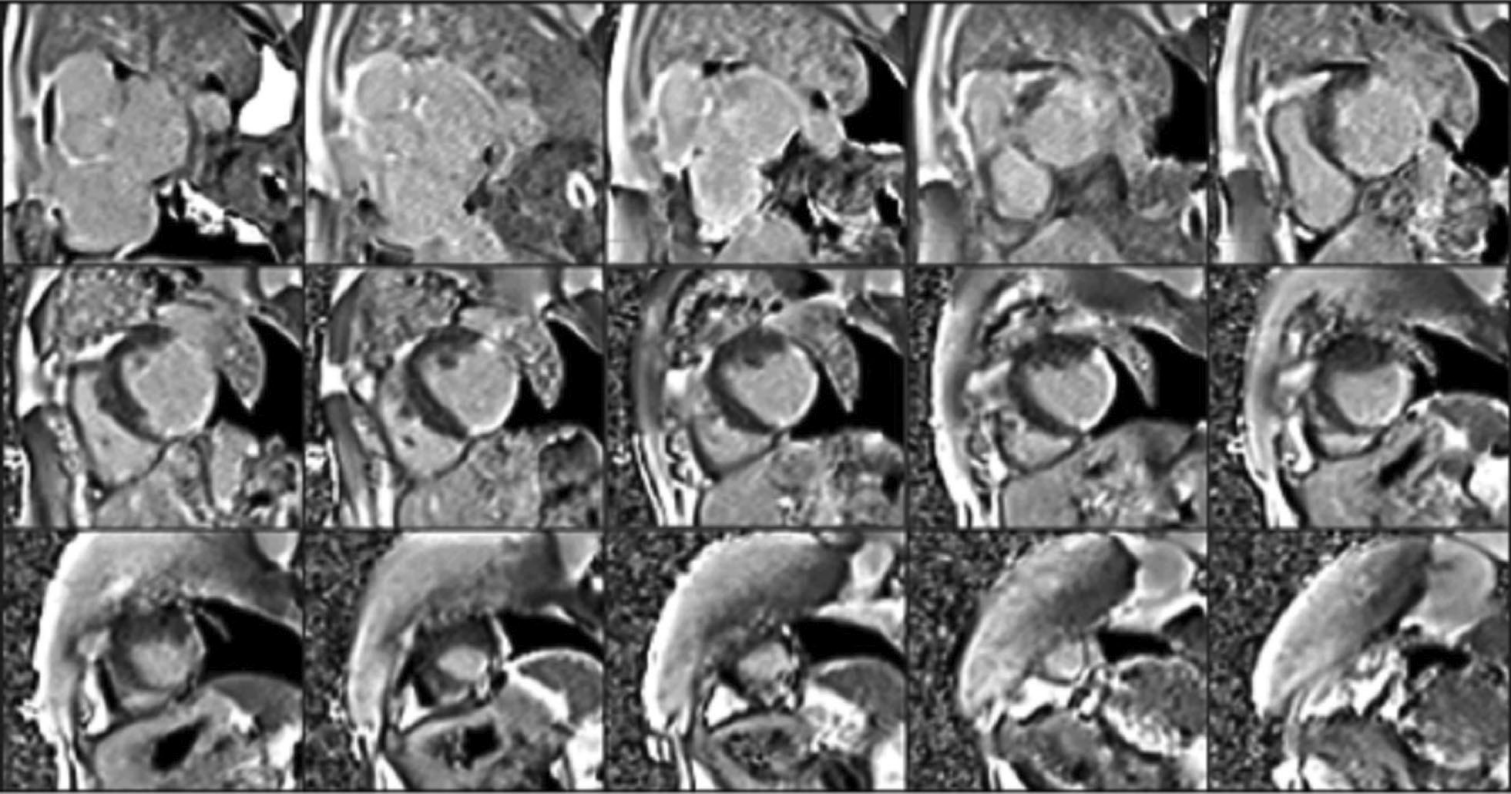
Case 4. Figure 4. LGE imaging demonstrating a large area of myocardial infarction with > 75% transmural extension in the territory of RCA and LCx

CMR was performed at 1.5 T (Aera, Siemens Healthineers AG, Erlangen, Germany). Cine imaging showed that the LV was severely dilated (LVEDVI 130 mL/m2) and systolic function was severely decreased (LVEF 34%)(Additional file 8: Case 4 Movie 1). In the territories of RCA and LCx, there was a large area of thinned LV wall associated with severe hypokinesis and akinesis, and LGE of > 75% transmural extent (Fig. 17 Case 4 Fig. 4). The apical cap was thin and aneurysmal, with signs of a transmural MI. There was moderate mitral regurgitation (regurgitation volume was 18 ml and regurgitation fraction was 34%). Peak aortic valve velocity was 3.1 m/s, which was compatible with moderate AS. Aortic regurgitation was mild.

### Conclusion

A large area in the RCA and LCx territories was thinned with significant transmural MI, and thus was considered not viable. AS was at least moderate and MR was moderate. Based on the lack of viability of a large myocardial territory, the risk/benefit for a surgical intervention with CABG along with valve(s) replacement was deemed unfavourable. The structural heart disease team considered her not to be a good candidate for transcatheter mitral valve replacement (TMVR). The option of transcatheter aortic valve replacement (TAVR) has been offered but patient decided not to pursue it.

The option of percutaneous coronary intervention (PCI) for the LCx and LAD disease was considered. However, revascularization of the presumed non-viable territory perfused by the small LCx was deemed to be of questionable value. PCI of the LAD was considered very challenging due to anticipated difficulty of wiring the diagonal branch and the associated concern of occluding it with stenting only of the LAD. In addition, the clinical impression was that her presentation was related to a significant degree to her valve disease(s) and addressing just the myocardial ischemia had an uncertain risk/benefit ratio. Her medical therapy was optimized by adding low dose losartan and ivabradine as the low dose metoprolol had to be held due to hypotension. At 2-week outpatient follow up she had adequate symptomatic control, albeit at a low, but acceptable for the patient, level of physical activity.

### Perspective

This case emphasizes the critical role of CMR in decision making for patient’s clinical management. We presented a case with heart failure related to non-ST-elevation myocardial infarction (NSTEMI) in the setting of coexisting AS and MR. Based on the results of TTE and cardiac catheterization, high-risk CABG and AVR were considered. However, CMR clarified the complex clinical picture leading to a unanimous agreement among the members of the heart team regarding the viable options for patient’s management, and possibility of TMVR was entertained instead of CABG and AVR. The absence of myocardial viability in 2 of the 3 coronary territories for which revascularization was planned, in addition to the other concerning clinical features (especially her frailty), resulted in the surgical team considering the patient too high risk for CABG and AVR.

Transmural extent of LGE is a well-established method to predict reversibility of myocardial dysfunction[29]. Especially in patients with high risk for surgery, CMR has been frequently utilized to select patients who are most likely to benefit from CABG. In patients with ischemic cardiomyopathy with LVEF ≤ 35%, combination of CABG and medical therapy has been shown to improve long-term mortality compared with medical therapy alone[30]. The applicability of the results of the STITCH trial in clinical practice is not always straightforward, as coronary revascularization appears to benefit patients with and without myocardial viability. However, patients without significant myocardial viability are at higher risk irrespective of the treatment strategy. In PARR2 study, there were lower cardiac events in patients with LV dysfunction using positron emission tomography (PET)-assisted management based on the amount of viable myocardium compared to standard care, however the results did not reach statistical significance [31]. The lack in significance was considered to be due to low adherence to the PET-assisted recommendations.

CMR has been considered as the gold standard in assessment of ventricular and atrial volume and function [32, 33]. There are increasing number of studies evaluating the reliability and accuracy of CMR in valvular heart disease. In AS, although TTE remains the gold standard for valve assessment, CMR phase contrast imaging provides reproducible results that are concordant with TTE[34]. Using bSSFP and phase contrast imaging, CMR quantification of MR has been demonstrated to be reliable as the difference between LV stroke volume and forward stroke volume[35]. It has been reported that CMR measures mitral regurgitation less severe than TTE does[35, 36]. Furthermore, post-surgical LV remodeling correlates well with CMR-assessed mitral regurgitation severity but not with TTE assessment, which is indicative of more accurate CMR assessment [35].

Management of this case was complicated by coexistence of both AS and MR in addition to ischemic cardiomyopathy. Per the guideline for the management of patients with valvular heart disease, AVR is a class 2b recommendation for patients with moderate AS who are undergoing cardiac surgery [36]. In the absence of surgical coronary revascularization, secondary mitral regurgitation of moderate severity is generally not a surgical indication per se. However, the management of co-existence of the three disease processes (e.g. coronary artery disease (CAD), AS, and mitral regurgitation) is not well defined, and is managed on a case-by-case basis.

The CMR of Case 4 (Additional file CMR Link, https://www.cloudcmr.com/6257-1973-9318-0151/).

## SCMR COVID-19 case collection

### Case 5: CMR findings in COVID-19 associated myocarditis

#### Clinical history

A 36 year old woman was admitted with pleuritic chest pain in the context of an acute COVID-19 infection. She was diagnosed with severe acute respiratory syndrome coronavirus 2 (SARS-CoV-2) by a positive reverse transcriptase PCR. Troponin-I was elevated at 13 ng/mL, and the D-dimer and other inflammatory biomarkers were elevated. She had a normal ECG with normal sinus rhythm, normal intervals, and no ST-T wave changes. TTE showed moderately decreased LV systolic function with global hypokinesis and mild RV dilation and mildly decreased systolic function.

#### CMR findings

**Fig. 18:**
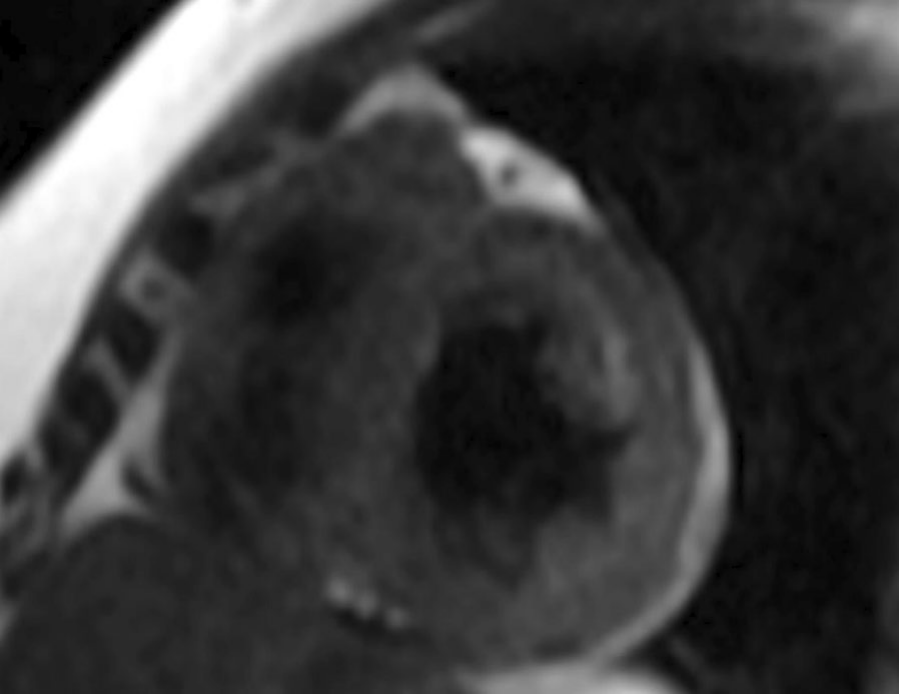
Case 5. Figure 1. T2 spin echo mid short axis. Increased signal in the lateral wall is present

**Fig. 19:**
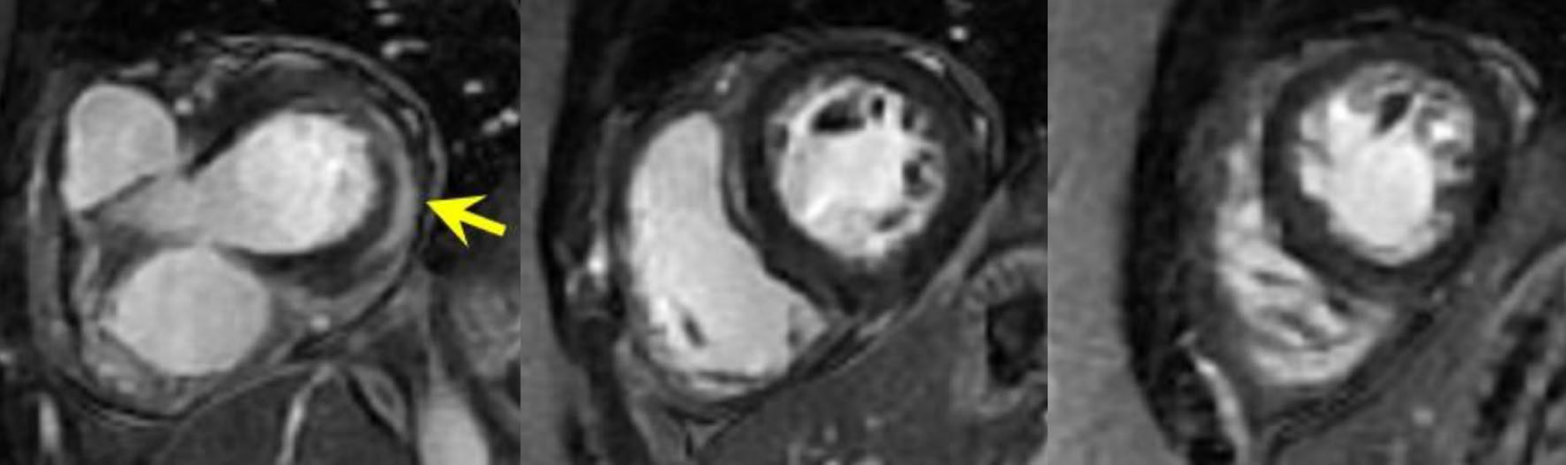
Case 5. Figure 2. Short axis stack myocardial delayed enhancement. There is basal lateral wall epicardial LGE (yellow arrow)

Moderately reduced LV systolic function (LVEF = 38%, Additional file 9: Case 5 Movie 1) with associated elevated T2 myocardial signal (Fig. 18 Case 5 Fig. 1) and epicardial LGE (Fig. 19 Case 5 Fig. 2) was present. The native global myocardial T1 value was 1204 ms (3 T General Electric Healthcare, modified Look-Locker inversion recovery sequence) and the global extracellular volume (ECV) was 28.4%. T1 and T2 color maps were not available.

#### Conclusion

CMR findings were consistent with myocarditis in the setting of COVID-19 infection. She was treated with intravenous methylprednisolone for two days followed by a rapid prednisone taper.

## Case 6: Cardiomyopathy in setting of COVID-19 infection

### Clinical history

**Fig. 20:**
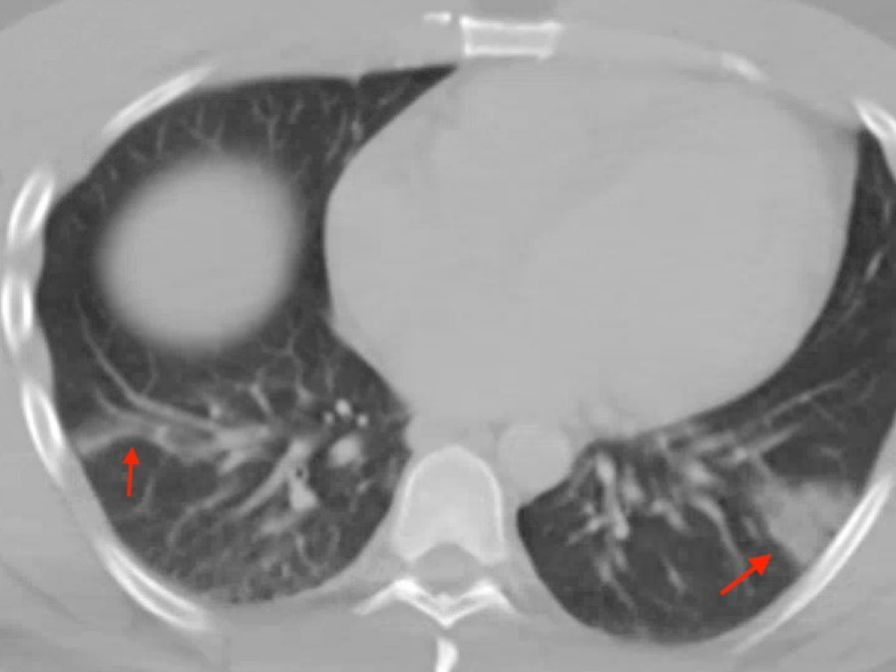
Case 6. Figure 1. Axial chest computed tomography (CT). Bilateral pulmonary consolidations (red arrows) present

**Fig. 21:**
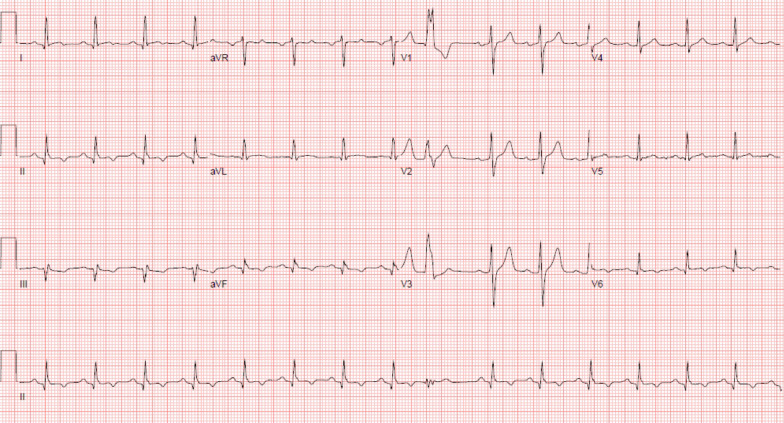
Case 6. Figure 2. Twelve lead ECG. Inverted T-waves in inferior leads and ventricular ectopy present

**Fig. 22:**
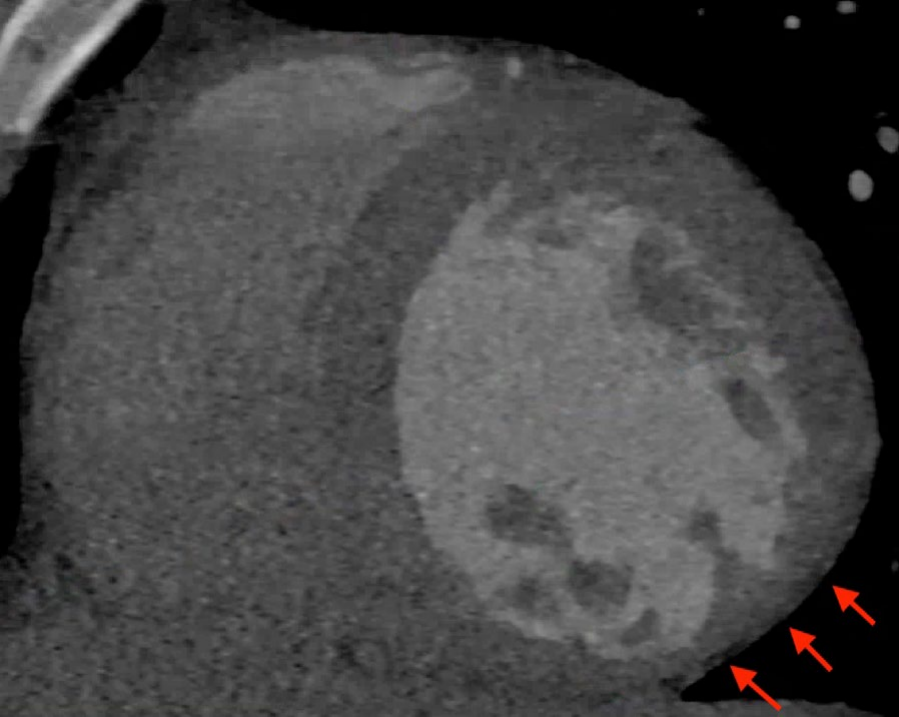
Case 6. Figure 3. CT coronary angiogram. LV inferolateral myocardial wall thinning (red arrows) present

A 41-year-old man with nonischemic cardiomyopathy (exercise single photon emission computed tomography (SPECT) thirteen years prior revealed no perfusion abnormalities and LVEF 44%) presented with fever, myalgias, abdominal pain, and shortness of breath. CT abdomen/pelvis with contrast was performed revealing no acute abnormalities in the abdomen but was notable for focal peripheral consolidation in the lungs (Fig. 20 Case 6 Fig. 1). The patient was treated for community-acquired pneumonia with ceftriaxone and prescribed a course of azithromycin upon discharge. He presented again one week later after having two syncopal episodes. Labs on admission were notable for leukopenia (WBC 3.56 K/uL, Ref 4.0–11.0 K/uL), elevated C-reactive protein (CRP) 44.2 mg/L (Ref 0–8 mg/L), high sensitivity Troponin T 100 ng/L (Ref 0–14 ng/L). SARS-CoV-2 PCR from nasopharyngeal swab was positive. ECG revealed normal sinus rhythm with occasional premature ventricular contractions (PVCs) with Q waves and T wave inversions in inferior leads (Fig. 21 Case 6 Fig. 2). An ischemic evaluation was pursued with coronary CT angiogram that revealed no coronary plaque or stenosis but was notable for myocardial thinning involving the basal and mid inferior wall of the LV (Fig. 22 Case 6 Fig. 3).

### CMR findings

**Fig. 23:**
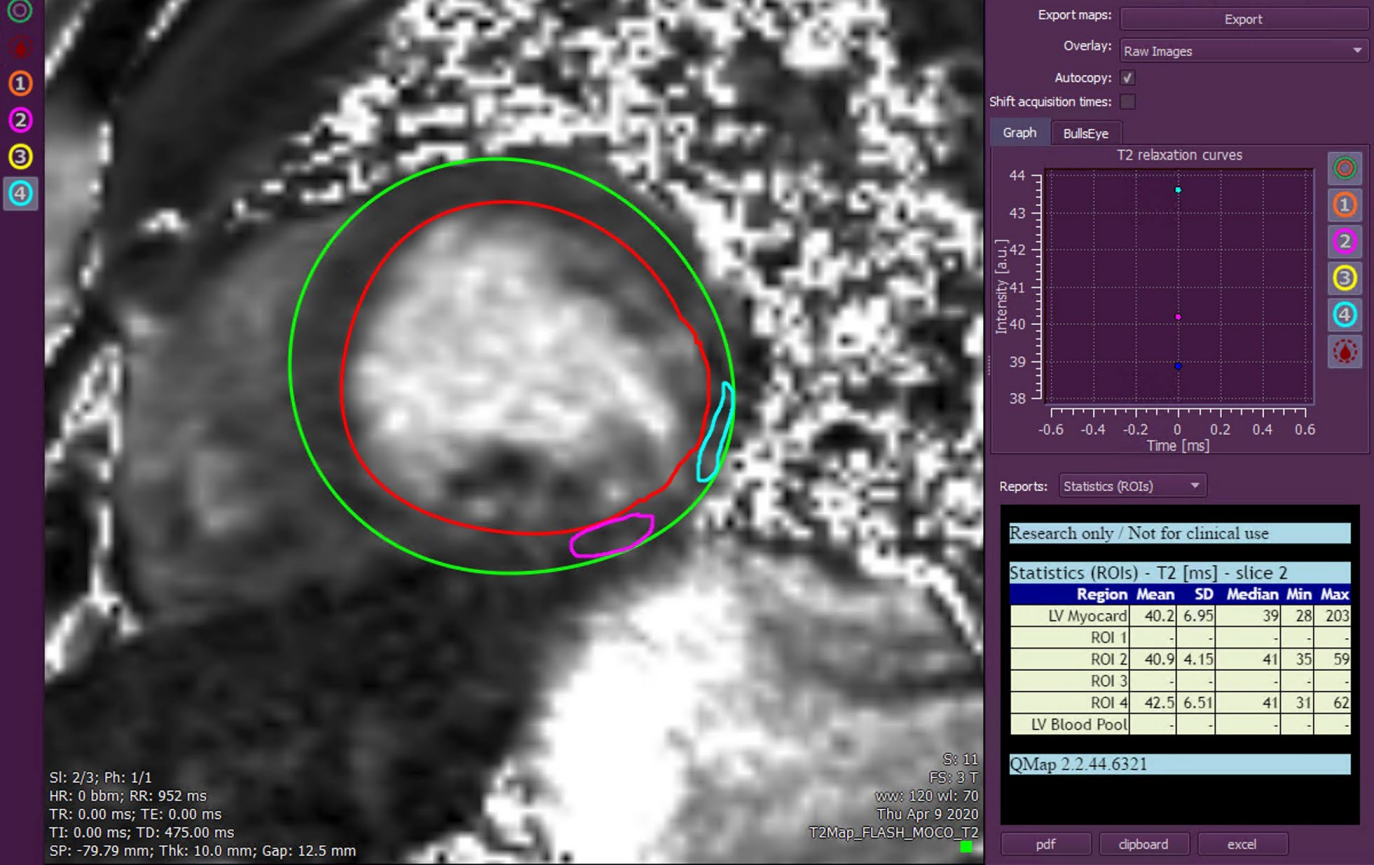
Case 6. Figure 4. T2 weighted mid short axis image. Semi-quantitative analysis showing a mean global myocardial T2 signal of 40 ms

**Fig. 24:**
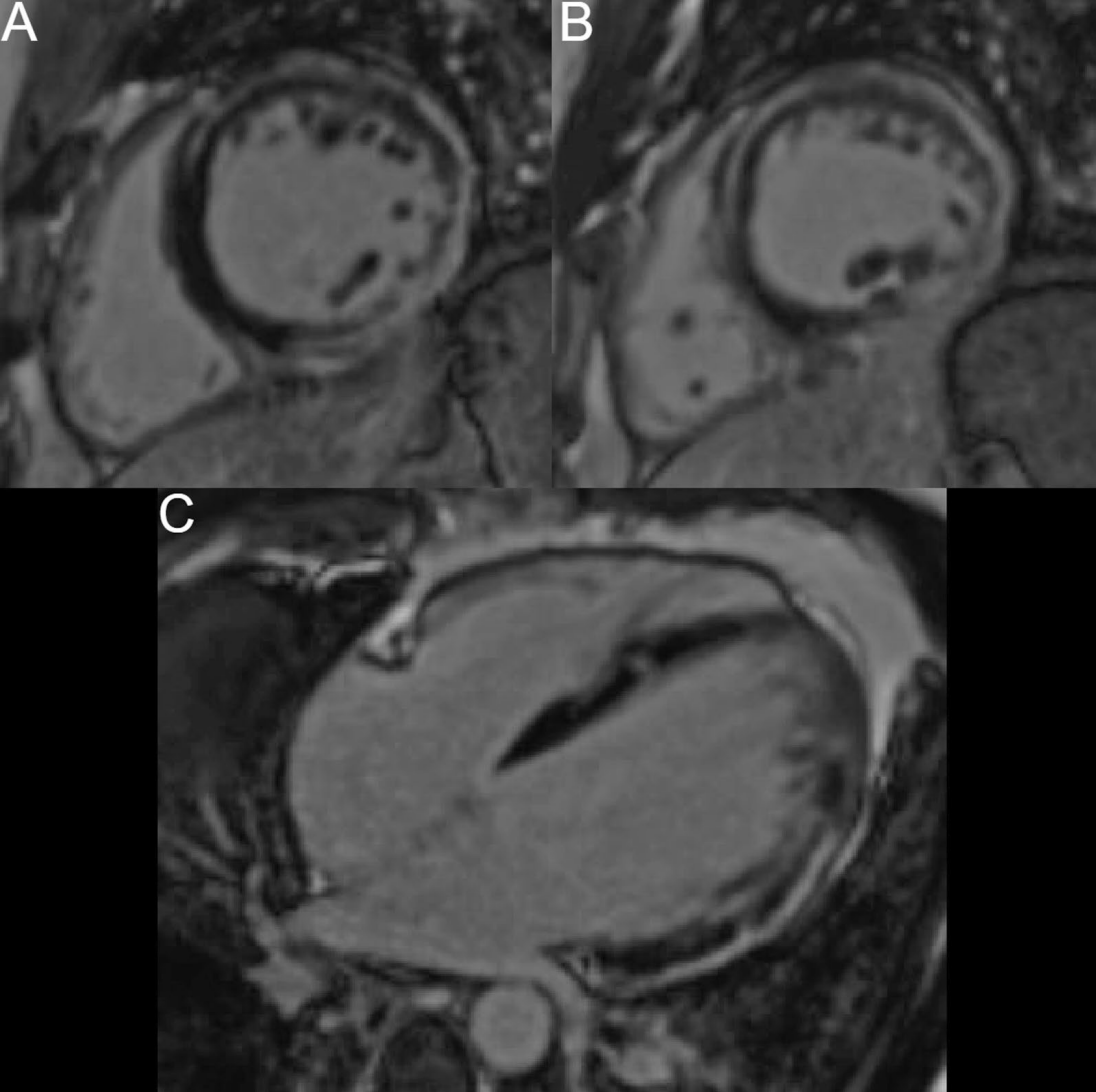
Case 6. Figure 5. Base **A** and mid **B** short axis and four chambr **C**) LGE. Epicardial enhancement involving the anterior, inferior, lateral and mid septal walls

CMR was obtained to evaluate for acute myocarditis. Cine bSSFP images revealed mildly dilated LV with moderately decreased global systolic function with LVEF 33% and dyskinesis of the inferolateral wall (Additional file 10: Case 6 Movie 1). T2 weighted imaging and T2 mapping did not demonstrate edema (Fig. 23 Case 6 Fig. 4). There was extensive, epicardial LGE involving the entire anterior, inferior, and lateral walls, and mid anteroseptal wall (Fig. 24 Case 6 Fig. 5).

### Conclusion

Overall, these CMR findings are consistent with cardiomyopathy with a non-ischemic pattern of fibrosis. Genetic testing would be a helpful next step in the evaluation of this cardiomyopathy. There is no evidence of acute myocardial edema based on T2 weighted imaging or T2 mapping. This case illustrates a part of the spectrum of clinical scenarios for which CMR is requested during the COVID-19 pandemic. CMR can evaluate the acuteness and etiology of cardiomyopathy or suspected acute coronary syndromes in patients presenting with cardiac symptoms suspected to be related to COVID-19 infection.

Goal-directed medical therapy for heart failure was initiated including beta-blocker and angiotensin converting enzyme inhibitor. The patient was discharged with a wearable external defibrillator and was then readmitted a few days later after presenting with multiple episodes of monomorphic VT requiring electrical cardioversion. He subsequently underwent implantation of dual-chamber ICD for secondary prevention of sudden cardiac death and was discharged on amiodarone for VT. ICD was interrogated three weeks later without any evidence of recurrent arrhythmias.

## Case 7: CMR findings in a young patient with frequent PVCs in setting of prior COVID-19 infection

### Clinical history

**Fig. 25:**
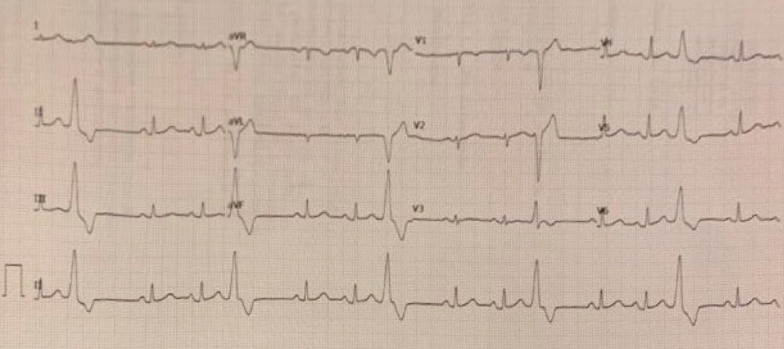
Case 7. Figure 1. Twelve lead ECG. Normal sinus rhythm without ST-T abnormalities and frequent premature ventricular contractions present

A 29-year-old woman was admitted for palpitations and fatigue. One month prior to admission, she had an upper respiratory infection characterized by cough, fever, and muscular pain. At that time, she did not seek medical attention and only took pain relievers. One week after the initial onset of symptoms, she developed mild diffuse chest pain and palpitations that persisted for two weeks. The 12 lead ECG showed sinus rhythm without any ST and T wave abnormalities and the presence of frequent monofocal PVCs (Fig. 25 Case 7 Fig. 1). Labs on admission were notable for mild elevated NT-pro BNP 144 (Ref 0–100 pg/ml) and normal troponin I 0.1 pg/ml (Ref 0–15 pg/ml), creatine kinase-MB 0.5 ng/ml (Ref 0–3,4 ng/ml) and D-dimer 191 ng/ml (Ref 0–250 ng/ml). SARS-CoV-2 PCR from nasopharyngeal swab was negative. In addition, she was with IgM-negative and IgG-positive for SARS-CoV-2 related prior infection.

### CMR findings

**Fig. 26:**
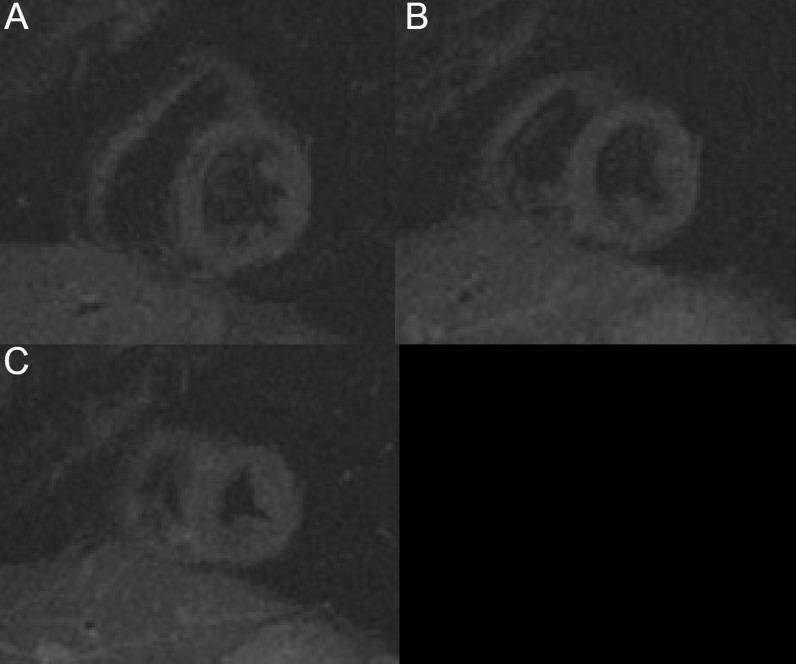
Case 7. Figure 2. Basal to apical (panels** A**-basal,** B**-mid, and** C**-apical respectively) shot axis short tau inversion recovery images. No hyperintensity regions present indicating absent myocardial edema

**Fig. 27:**
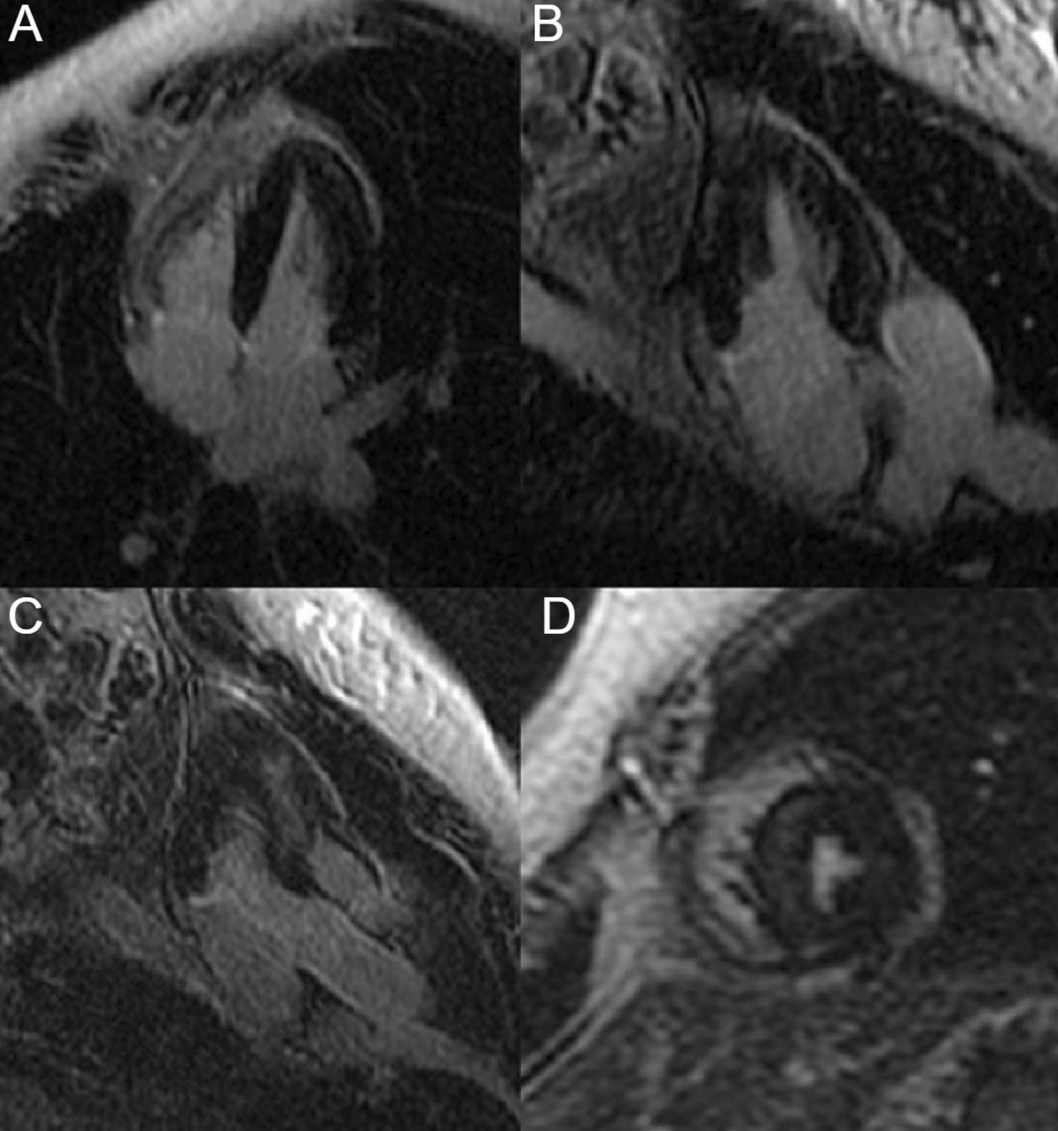
Case 7. Figure 3. Four chamber (**A**), two chamber (**B**), three chamber (**C**), and mid short axis **D** LGE images. Likely patchy mid-myocardial LGE affecting the mid anterior and mid to apical anteroseptal walls. No pericardial enhancement present

CMR at 1.5 T was performed one month after the onset of symptoms. There were no specific pulmonary abnormalities with no pleural or pericardial effusion (Additional file 11: Case 7 Movie 1). Image quality was affected due to the presence of frequent ventricular arrhythmia throughout the study. Long axis views of both ventricles showed normal ventricular volumes, wall thickness, ventricular mass, and ejection fraction. (Additional file 12: Case 7 Movie 2). The left and right ventricular volumes were as follows: LVEDVI 40.1 ml/m2, LVESVI 14.6 ml/m2, LVEF 63%, RVEDVI 38.2 ml/m2, RVESVI 17.2 ml/m2, RVEF 55%. Myocardial edema was absent on T2 STIR images (Fig. 26 Case 7 Fig. 2) with the ratio between myocardial/body muscle normal at 1.8 (Normal value < 2). LGE sequences showed extensive, patchy intramyocardial enhancement affecting the mid anterior and mid to apical anteroseptal walls. (Fig. 27 Case 7 Fig. 3, Additional file 13: Case 7 Movie 3).

### Conclusion

The patient was discharged five days after hospital admission with antiarrhythmic medication and close follow-up. A future CMR is planned in 6 weeks for surveillance of myocardial fibrosis and its relationship with ventricular arrhythmia. This case provides evidence of CMR sequelae of likely prior myocardial inflammatory involvement possibly secondary to SARS-CoV-2 infection that has been described in some patients. The absence of edema with the persistence of LGE may not be reversible. LGE images could have some difficulties during acquisition and a low inversion time can suggest hyperenhancement even in the absence of fibrosis with a need to be confirmed in imaging surveillance. Additionally, further investigations are required to clarify these findings and clinical relevance in future arrhythmic events of these patients.

## Case 8: The role of multimodality imaging during a severe COVID-19 infection

### Clinical history

A 60-year-old male volunteer for the homeless presented to the emergency room with a two week history of progressively worsening shortness of breath. He had been diagnosed with COVID-19 by a positive SARS-CoV-2 PCR in an urgent care facility 4 days prior to presentation. Other associated symptoms included intermittent fever, malaise, and cough. He had noted that his O_2_ saturations dropped as low as 75% on his home pulse oximeter. He had no significant past medical history and was very active prior to this episode.

**Fig. 28:**
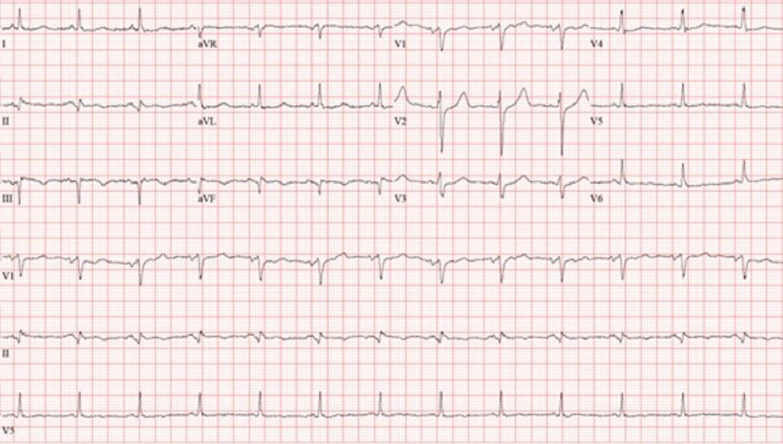
Case 8. Figure 1. Twelve lead ECG. Sinus rhythm present with Q waves in the inferior leads (II, III and aVF)

In the emergency room, he was afebrile, blood pressure was 110/74 mm Hg, heart rate 94 bpm and O_2_ saturation was 87% on room air. On physical exam, there were mild crackles on the lower lung fields bilaterally. ECG (Fig. 28 Case 8 Fig. 1) showed sinus rhythm with Q waves in the inferior leads (II, III and aVF).

**Fig. 29:**
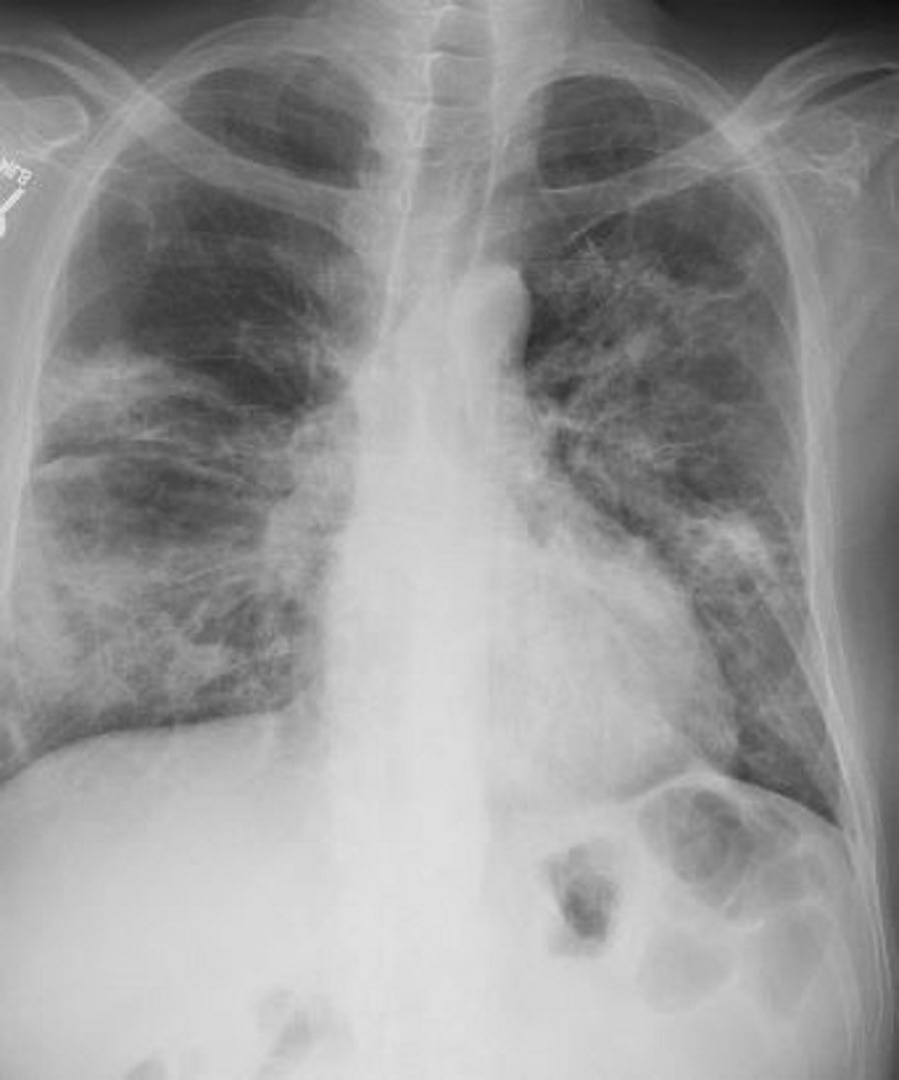
Case 8. Figure 2. Chest X-ray. Multifocal interstitial and patchy alveolar airspace opacities noted throughout the mid right lower lung and mid left lung

Pertinent laboratory findings included an elevated white blood cell count 11,300 cells/mcL, elevated D-dimer 15,477 ng/mL, elevated lactate dehydrogenase 498 unit/L, elevated NT-pro BNP 199 pg/mL, minimally elevated troponin 0.06 ng/mL, elevated ferritin 2,342 ng/mL. He was empirically started on full dose heparin. Chest X-ray (Fig. 29 Case 8 Fig. 2) showed multifocal interstitial and patchy alveolar airspace opacities throughout the mid right lower lung and mid left lung consistent with multifocal infection, likely COVID-19.

**Fig. 30:**
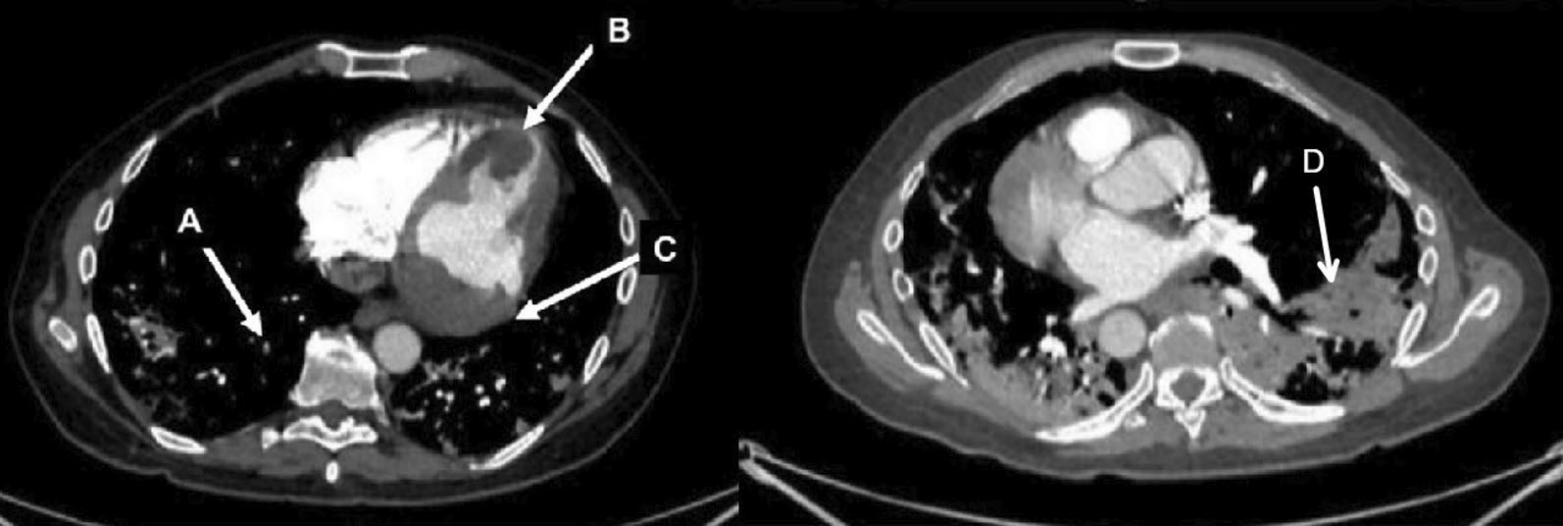
Case 8. Figure 3. CT scan of the chest. Contrast axial view showing a sub-segmental pulmonary embolism of the right lower lobe (**A**), a mass near in the inferior border of the heart **B** and in LV apex (**C**), and multifocal pulmonary infiltrates (**D**)

**Fig. 31:**
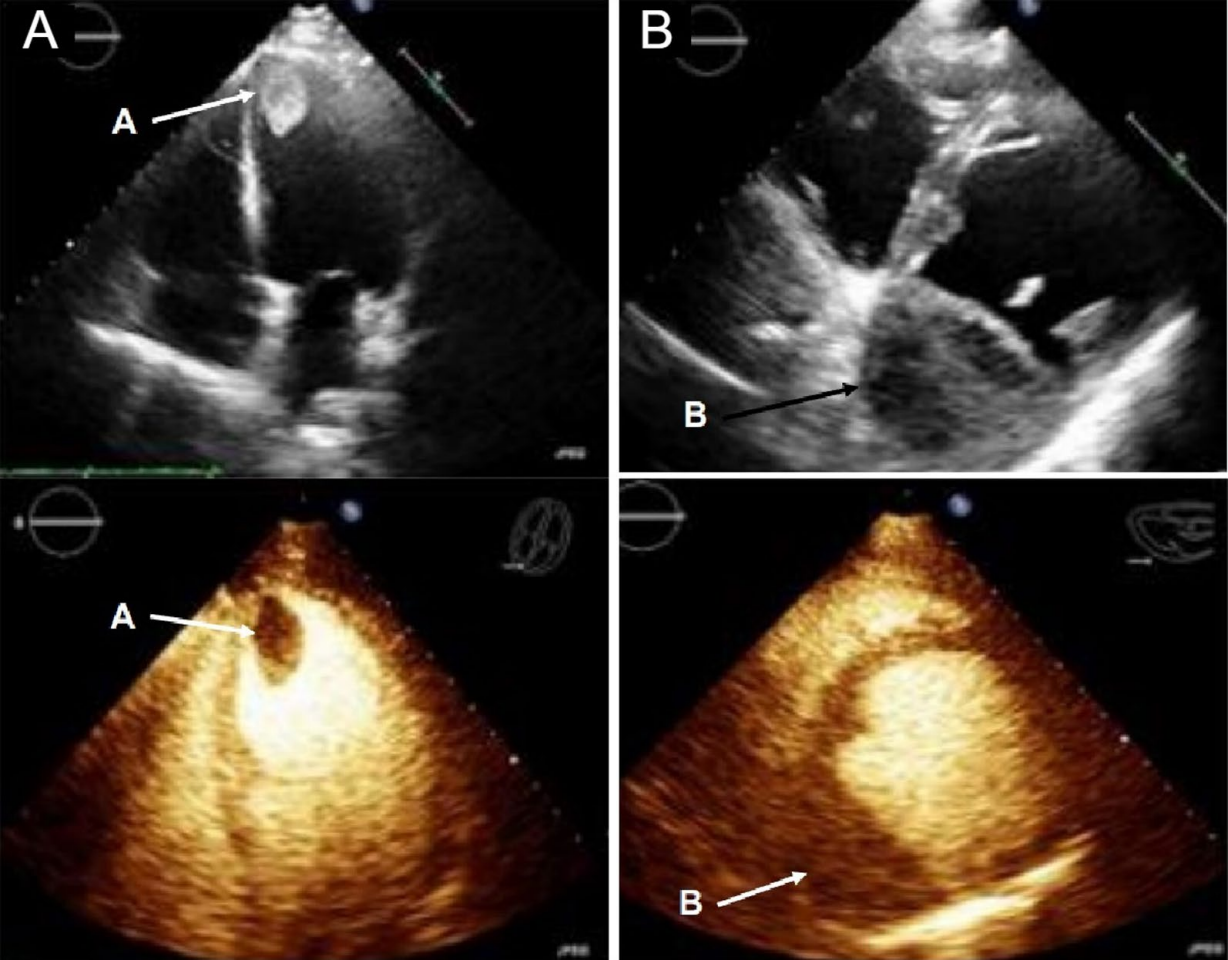
Case 8. Figure 4. Transthoracic echocardiogram (TTE) four chamber **A** and short axis **B** views. Non contrast and contrast enhanced views showed the presence of the masses in the LV apex **A** and inferior wall (**B**)

Chest CT with contrast (Fig. 30 Case 8 Fig. 3) showed a sub-segmental pulmonary embolism of the right lower lobe without evidence of heart strain or pulmonary infarction. Lung parenchymal findings consistent with COVID-19 pneumonia. There was an expansile rounded soft tissue density along the inferior LV border and another mass at the LV apex. A TTE with contrast (Additional file 14: Case 8 Movie 1, Fig. 31 Case 8 Fig. 4) confirmed cardiomyopathy with multiple wall motion abnormalities as well as the presence of the masses in the LV. A CMR was recommended for further evaluation.

### CMR findings

**Fig. 32:**
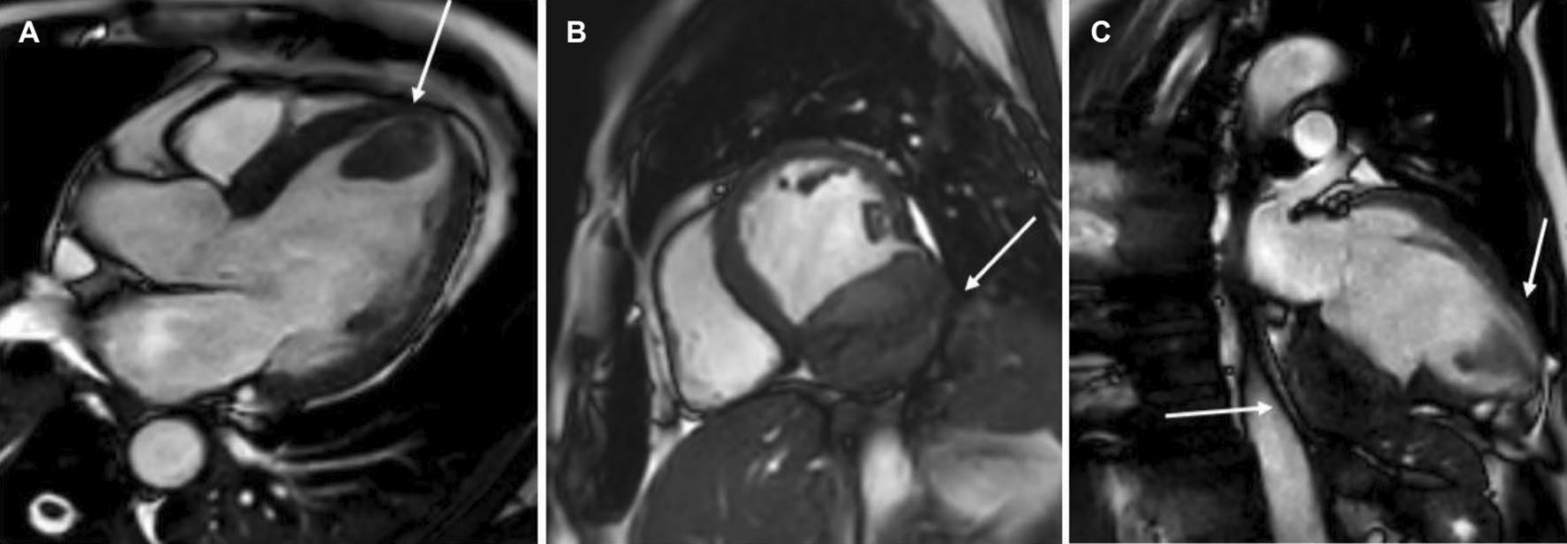
Case 8. Figure 5. Cine bSSFP three chamber (**A**), short axis (**B**), and two chamber (**C**) views. Masses (arrows) present in the LV apex and inferior wall

**Fig. 33:**
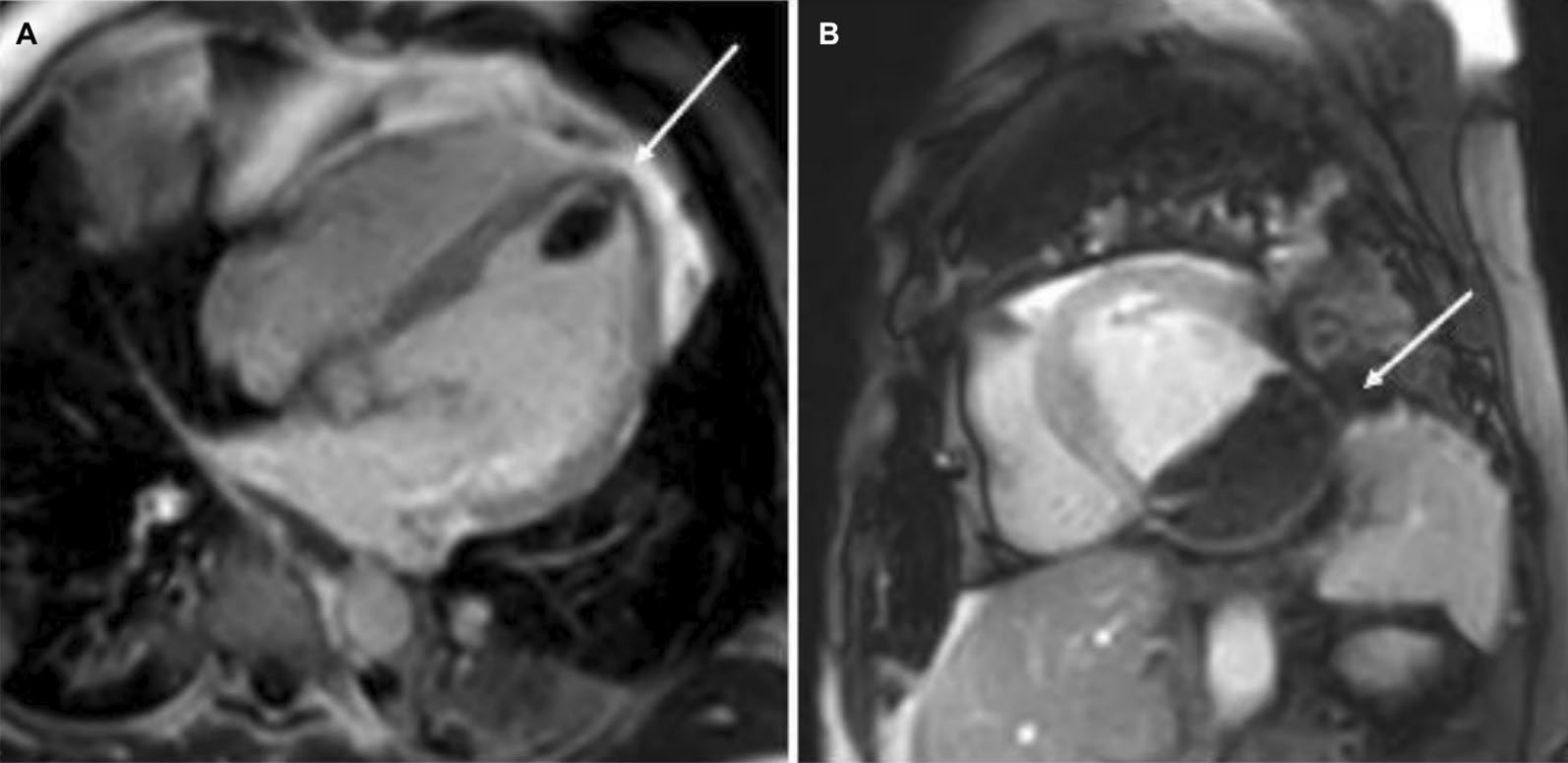
Case 8. Figure 6. First pass perfusion four chamber **A** and short axis **B** views. No uptake of contrast present in either masses (arrows)

The patient was scanned on a 1.5 T scanner using a ‘cardiac mass’ protocol. The bSSFP cine images revealed that the LV was severely dilated with severely reduced systolic function. The LVEF was 23%. The basal and mid inferior and inferolateral walls were thin, aneurysmal and occupied by a large mass measuring 6.1 × 4.4 cm. The entire apex was thin, akinetic, and there was another mass measuring 3.5 × 1.8 in the LV apex (Additional file 15: Case 8 Movie 2, Fig. 32 Case 8 Fig. 5). First pass rest perfusion images showed no uptake in either of the masses (Additional file 16: Case 8 Movie 3, Fig. 33 Case 8 Fig. 6).

**Fig. 34:**
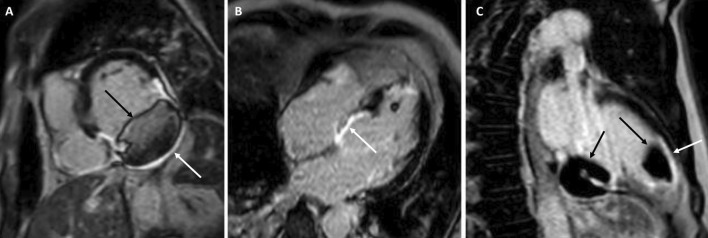
Case 8. Figure 7. LGE phase sensitive inversion recovery short axis (**A**), four chamber (**B**), and two chamber **C** views. Large transmural infarct present in the basal and mid inferior, inferoseptal and inferolateral walls as well as the inferior apical wall (white arrows) and 50% subendocardial enhancement consistent with infarct of the entire LV apex and the right ventricular (RV) inferior wall and apex (white arrows). No enhancement present of the masses (black arrows) of the inferior and apical walls

**Fig. 35:**
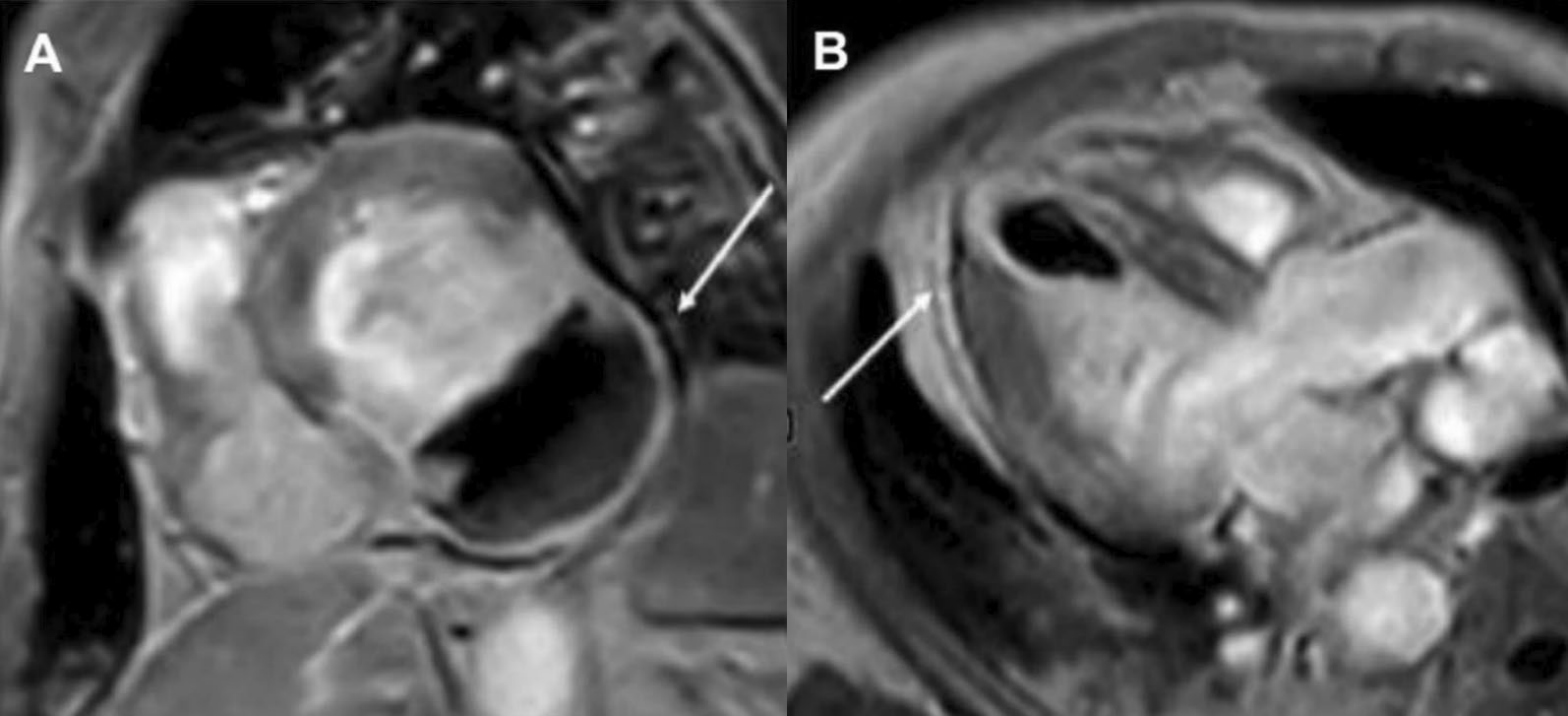
Case 8. Figure 8. LGE phase sensitive inversion recovery with long inversion time (600 ms) short axis **A** and three chamber **B** views. Thrombus is present in the inferior wall and apex (arrows)

LGE images showed a large transmural infarct in the basal and mid inferior, inferoseptal and inferolateral walls as well as the inferior apical wall. There was also a 50% subendocardial LGE region consistent with infarct of the entire LV apex and the RV inferior wall and apex (Fig. 34 Case 8 Fig. 7). Another set of delayed gadolinium images were scanned using a prolonged inversion time (i.e. 600 ms) which selectively nulls avascular tissue such as thrombus. This prolonged inversion time renders thrombus black and surrounding myocardium bright as shown in Fig. 35 Case 8 Fig. 8. This further corroborated the masses as being thrombi. He was continued on treatment for HF, intracardiac thrombi and pulmonary embolism. He was enrolled in a clinical trial for treatment of severe COVID-19 infection.

### Conclusion

In this case, it was thought that the patient likely had COVID-19 related coronary thrombosis of his RCA two weeks prior to presentation that caused an inferior MI. His severe COVID-19 infection caused a hypercoagulable state which led to both arterial and venous thromboemboli (the RCA was occluded on the detailed review CT pulmonary angiogram and the left sided coronary arteries were well visualized on the CT chest). The CMR facilitated in diagnosing the patient’s infarct and intracardiac thrombi. He was started on goal directed medical therapy for HF as well as anticoagulation therapy and discharged on COVID-19 clinical trial medication. He was scheduled to see a cardiologist in one-week post discharge at which time they would decide a further treatment plan.

## Case 9: A case of elusive recurrent COVID-19 pericarditis?

### Clinical history

A 52 year old male with no significant past medical history had been having intermittent fevers, chills, dry cough, left scapular and pleuritic chest pain associated with exertional dyspnea for more than one month. He had three prior hospitalizations since his symptoms began. He was initially diagnosed with right middle lobe pneumonia when hospitalized at an outside facility, treated with antibiotics and had subsequent mild symptom relief on discharge. He was hospitalized twice again for the same complaints at the outside institution. CT angiography of the chest was done on both admissions and showed no evidence for pulmonary embolism or pneumonia, but demonstrated a pericardial effusion and small left pleural effusion. His SARS-CoV-2 PCR test was negative but his SARS-CoV-2 antibody was positive. He was given a short course of antibiotics during one of those hospitalizations without benefit.

**Fig. 36:**
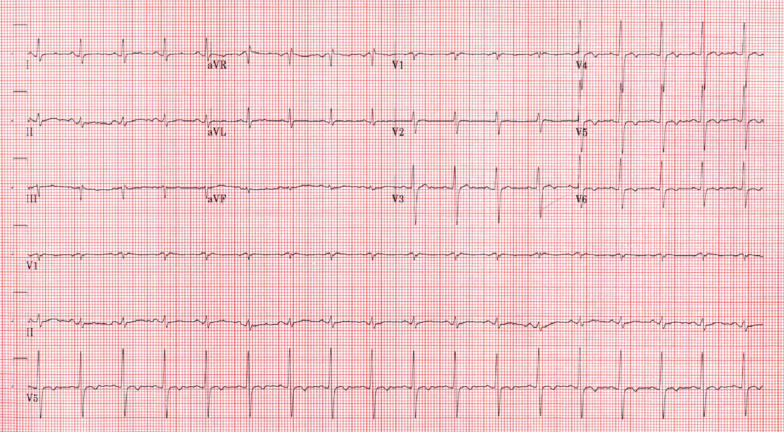
Case 9. Figure 1. Twelve lead ECG. Sinus tachycardia and non-specific ST abnormalities present

Despite previous treatments, he again had a relapse of symptoms and was admitted to our facility. Social history revealed his mother had contracted SARS-CoV-2 and died from COVID-19 pneumonia about 2 months prior to his presentation. He was febrile to 102.7F on admission with a mildly elevated white blood cell count of 11.5 K. Initial blood work showed elevated inflammatory markers (CRP 143 mg/L, sedimentation rate of 40 mm/hr) and mild troponin elevation to 0.1 ng/mL. Chest X ray showed clear lungs and chest CT did not show pneumonia. Blood cultures remained without growth. ECG showed sinus tachycardia with non-specific ST abnormalities in leads V4-V6 (Fig. 36 Case 9 Fig. 1). SARS-CoV-2 PCR was negative, however SARS-CoV-2 antibody was positive again.

TTE demonstrated moderately reduced LVEF of 35–40% with diffuse moderate hypokinesis, trivial circumferential pericardial effusion, and small left sided pleural effusion. CT coronary angiography was done to exclude obstructive coronary artery disease and revealed no significant coronary artery stenosis. However, there was notable pericardial thickening measuring about 0.6 cm but absent pericardial calcification. The patient was referred for CMR for further evaluation.

### CMR findings

**Fig. 37:**
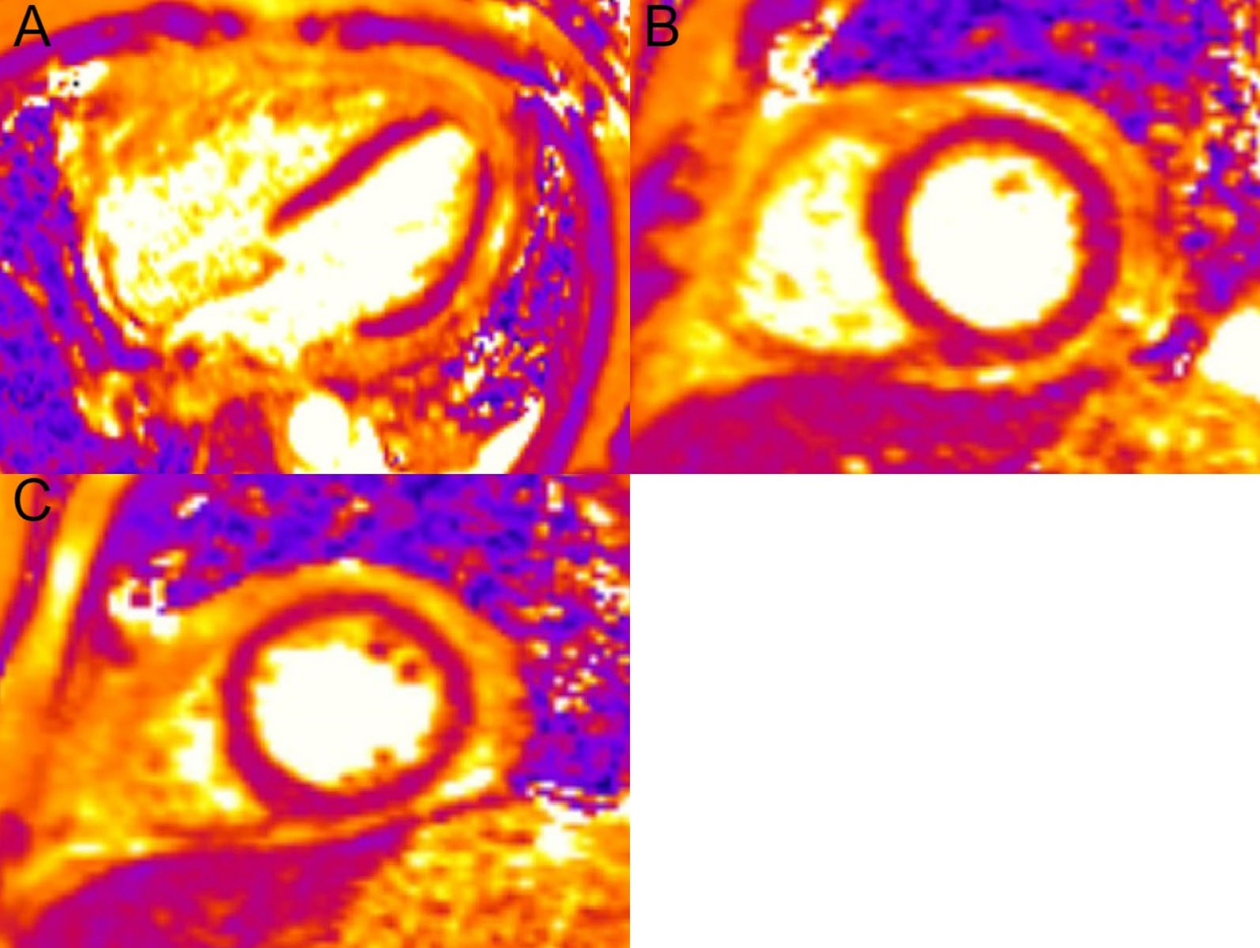
Case 9. Figure 2. Four chamber **A** and basal **B** and mid **C** short axis T2 maps. No evidence of myocardial inflammation or edema present

**Fig. 38:**
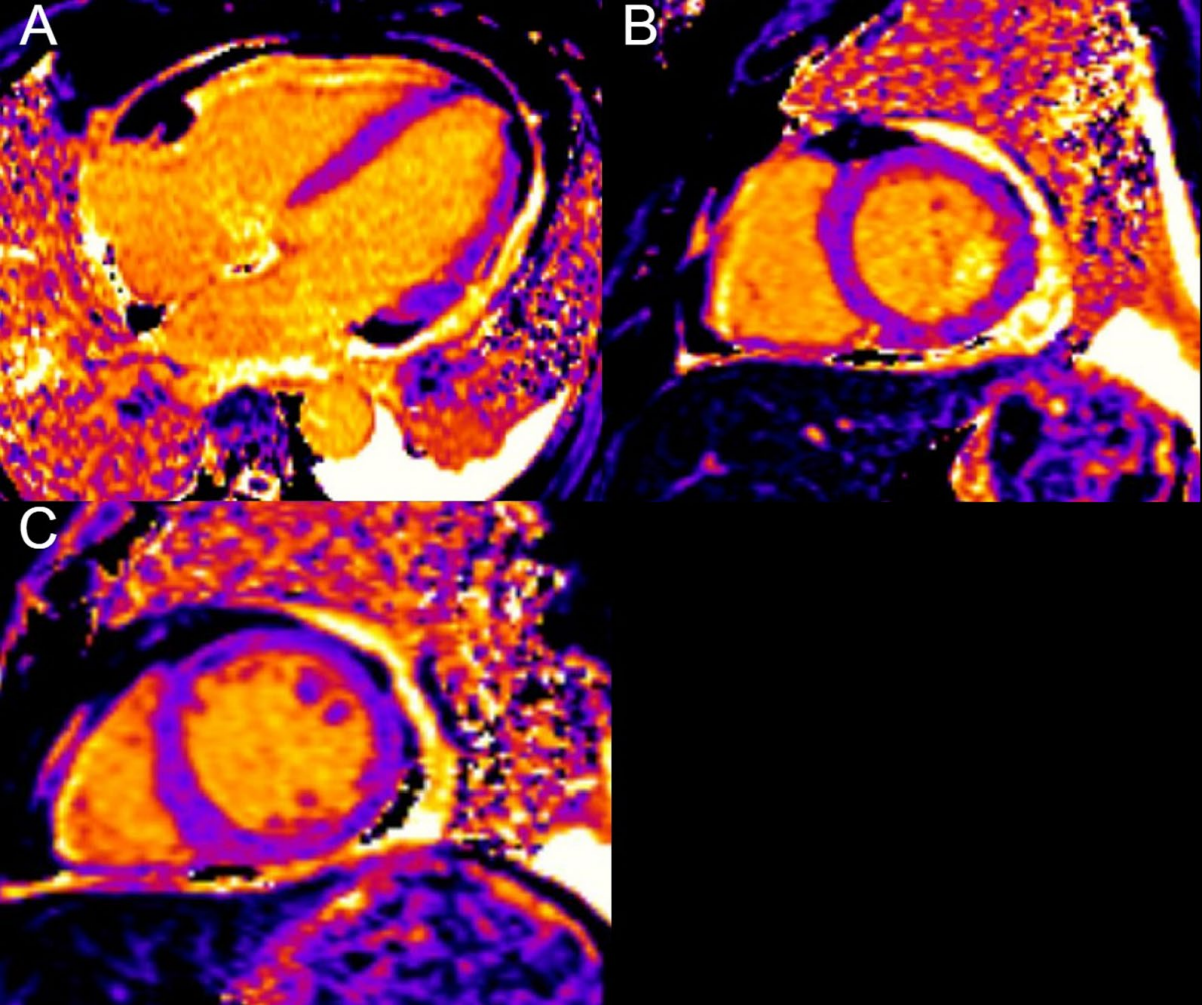
Case 9. Figure 3. Four chamber **A** and basal **B** and mid **C** short axis native T1 maps. Native T1 values were within normal reference institutional ranges

**Fig. 39:**
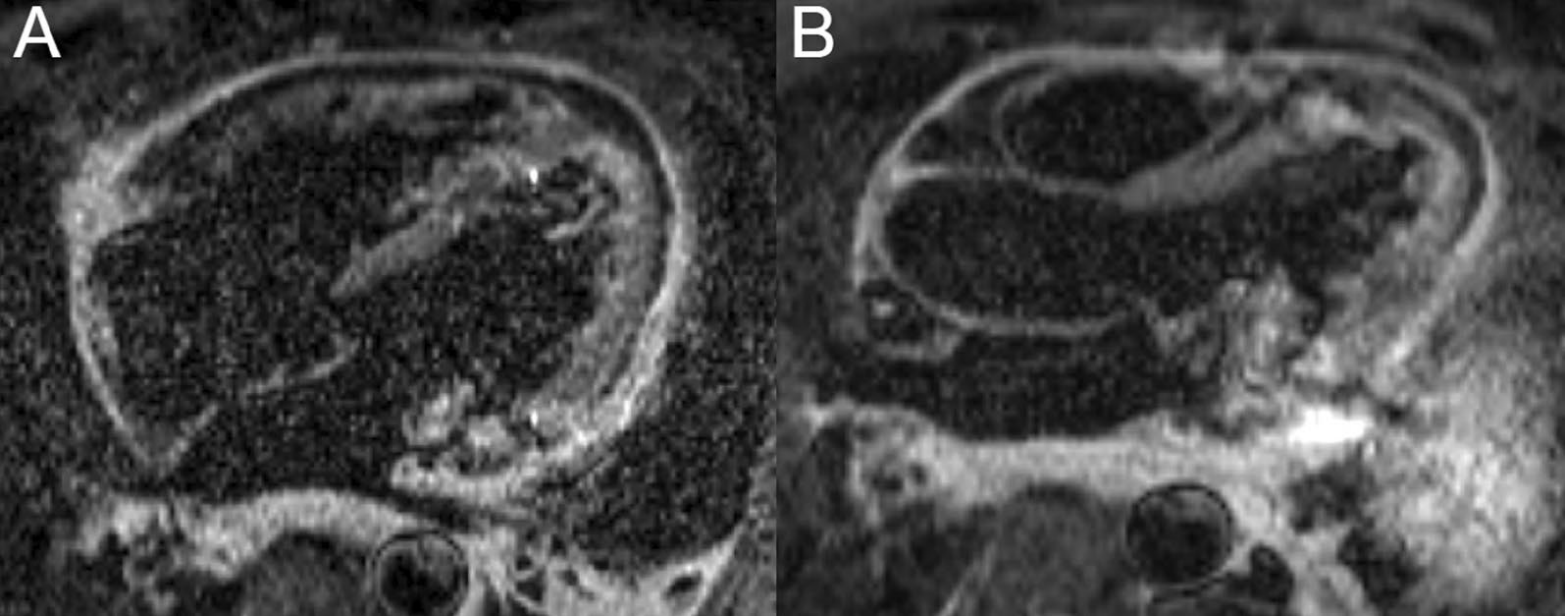
Case 9. Figure 4. Four **A** and three **B** chamber T2 short tau inversion recovery. Pericardial thickening and inflammation and edema present

**Fig. 40:**
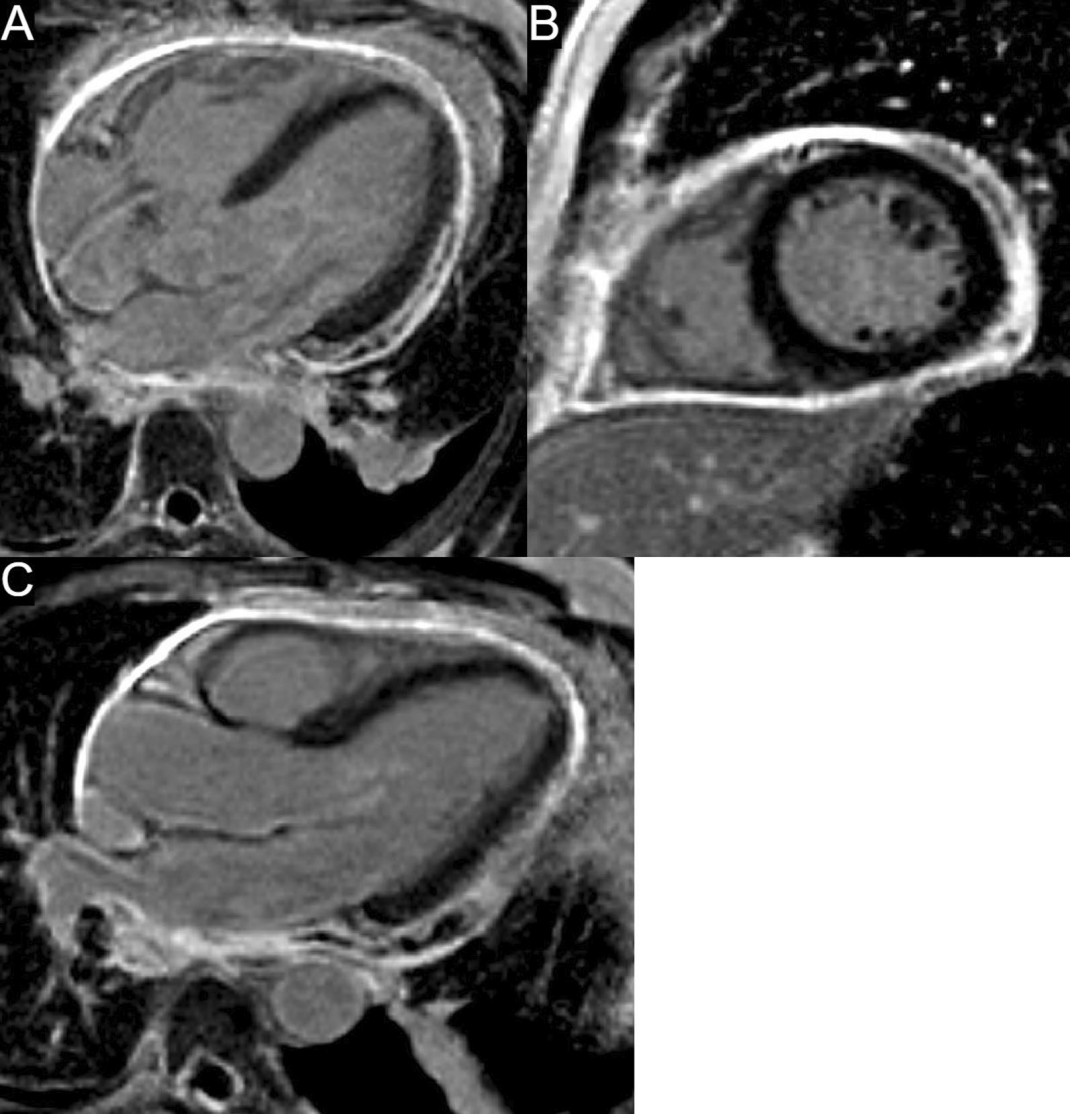
Case 9. Figure 5. Four chamber (**A**), short axis (**B**), and three chamber **C** post contrast inversion recovery gradient echo images. Marked LGE of the pericardium present

Biventricular systolic function was low normal with an LVEF of 52% and an RVEF of 50% (Additional file 17: Case 9 Movie 1). There was no evidence of myocardial inflammation or edema as manifested by normal myocardial T2 mapping values (Fig. 37 Case 9 Fig. 2). Native T1 values were within reference range for our institution, measuring 988 ms, averaged over 8 short axis segments (Fig. 38 Case 9 Fig. 3). Furthermore, the myocardial ECV was 24%. However, on T2 weighted STIR, high signal intensity and marked thickening (measuring 0.5 cm) of the pericardium was present (Fig. 39 Case 9 Fig. 4). There was also extensive pericardial LGE (Fig. 40 Case 9 Fig. 5). There was no mid wall or epicardial myocardial LGE to suggest sequelae of previous myocarditis. A trivial pericardial effusion was present with no evidence of interventricular dependence by free-breathing real time cardiac cine (Additional file 18: Case 9 Movie 2).

### Conclusion

With suspected COVID-19 related pericarditis identified, the patient was treated with colchicine. He was not treated with high dose non-steroidal anti-inflammatory agents or aspirin. He had no further fever while admitted and symptomatically improved at the time of discharge. SARS-CoV-2 antibody being positive and social history of his mother having suffered from COVID-19 supports exposure to COVID-19. A negative SARS-CoV-2 PCR supports no active infection. The patient’s symptoms were isolated, which excluded the diagnosis of multisystem inflammatory syndrome in adults (MIS-A). The patient had full recovery after treatment with colchicine and upon follow up 6 months later, he has remained asymptomatic and active.

## Perspective COVID-19 case collection

There have been several studies evaluating cardiovascular complications from COVID-19 early in the pandemic[37–39]. Myocarditis is inflammation of the myocardium and typically is caused by infectious diseases either from a direct viral infection or post-viral immune-mediated reaction[40]. Many different viruses are linked to myocarditis, and the most common viral pathogens have shifted over the decades[41]. CMR has greatly enhanced the ability to characterize myocardial inflammation in patients with suspected myocarditis and to predict all-cause cardiac mortality. CMR provides reproducible volumetric and functional data with the unique ability of localizing areas of myocardial inflammation and edema. Specific techniques for myocardial characterization have been developed including T2-weighted imaging to localize myocardial edema and T1-weighted images for hyperemic response[42, 43]. The use of LGE techniques has used to identify areas of myocardial injury (necrosis and fibrosis) and inflammation[40, 43, 44]. In the setting of clinically suspected myocarditis, the diagnostic CMR criteria (Lake Louise Consensus Criteria) for myocardial inflammation are present if at least two of the above mentioned myocardial tissue characterization parameters are present (edema, hyperemia, or fibrosis)[40]. The updated Lake Louise Criteria leveraged novel parametric mapping techniques including native T1, T2, and ECV mapping[45].

Several cases of myocarditis related to COVID-19 have been documented in the literature [46–49]. The prevalence of myocarditis in COVID-19 patients is unknown, but it has been reported that up to 7% of COVID-19 related deaths were attributable to myocarditis[50, 51]. Clinical presentation of COVID-19 associated myocarditis varies among cases as shown with this case series.

An evaluation of myocardial tissue characteristics by CMR in previously hospitalized patients with COVID-19 in China revealed 54% with myocardial edema (elevated T2) and 31% with myocardial fibrosis (positive LGE)[52]. All of these patients were previously hospitalized and had clinical symptoms consistent with myocarditis[52]. A study from Germany performed on 100 previously COVID-19 positive patients showed similar findings on CMR with 60% with myocardial edema (elevated T2) and 32% with myocardial fibrosis (positive LGE)[52, 53]. However, only 33% of this cohort required hospitalization, 18% were asymptomatic, and several patients enrolled had other comorbidities that could affect CMR parameters[53].

The pathogenesis of hypercoagulability has been commonly explained through Virchow’s triad of stasis, endothelial injury, and a hypercoagulable state. Severe infection with SARS-Co-2 virus causes a proinflammatory state and elevation of levels of several prothrombogenic factors[54]. The infection has also proven to cause direct endothelial cell injury as well as complement mediated damage. There are several studies that showed a higher incidence of venous thromboembolism (VTE) (both deep vein thrombosis and pulmonary embolism) in intensive care unit (ICU) and non-ICU patients (though to a lesser extent than ICU patients) infected with SARS-Co-2 virus[55, 56]. There are also several reports of arterial thrombosis including microvascular thrombi, limb ischemia and strokes. It is for these reasons that the American Society of Hematology and the Society of Critical Care Medicine recommend routine aggressive pharmacologic prophylaxis for VTE unless contraindicated.

Several studies have shown a strong association between risk factors for CAD such as diabetes, hypertension, prior CAD and the severity of COVID-19 infection [57]. Severe COVID-19 infections have shown to increase the risk of both Type 1 and type 2 MI the latter through hypoxia related illness due to respiratory compromise and primary infection [58]. Troponin elevation is seen in about 10 to 30% of hospitalized COVID-19 patients and was found to be associated with a higher mortality [59]. However, most of the patients presenting with COVID-19 infection, it was found that troponin elevation was not due to acute coronary syndrome (ACS) but secondary to a non-ACS type of myocardial injury such as myocarditis, pericarditis, heart failure, arrhythmia related or related to lung injury [59].

The importance of using CMR despite TTE findings in adult patients with COVID-19 is highlighted by Case 9. The patient’s TTE mistakenly demonstrated a LVEF of 35–40%, leading to inappropriate therapy with metoprolol and losartan for “acute systolic congestive heart failure”. Of note, the patient’s NT-pro BNP was 48 pg/mL with a non-obese BMI (26 kg/m2). The patient possibly had recurrent post infectious COVID-19 pericarditis as manifested by his symptom course and persistently positive SARS–CoV-2 antibody. He is also at high risk for further recurrence as manifested by pericardial LGE [60]. In the above ways, CMR drastically altered patient management.

## Supplementary Information

**Additional file 1. Case 1 Movie 1.** Transthoracic echocardiogram (TTE) parasternal long axis view. There was pericardial thickening and a circumferential pericardial effusion present.

**Additional file 2.** **Case 1 Movie 2.** Cine SSFP four chamber and mid short axis views. There was normal biventricular systolic function, a moderate circumferential pericardial effusion, brief right atrial (RA) compression, and mild pericardial thickening present.

**Additional file 3.** **Case 2 Movie 1.** Four **A** and two chamber **B** cine bSSFP images. Septal and anterior LV hypertrophy and myocardial crypts of the basal anterior and anterolateral walls.

**Additional file 4.** **Case 3 Movie 1.** Apical four chamber TTE. There is normal LV systolic function, mild tricuspid valve insufficiency, and the right ventricle is not well seen.

**Additional file 5.** **Case 3 Movie 2.** Four chamber cine bSSFP stack. Dilated RV with RV dyskinesia and irregularity noted in the LV lateral wall.

**Additional file 6.** **Case 3 Movie 3.** Two chamber right **A** and left **B** cine bSSFP. RV microaneurysms along the free wall consistent with the “accordion sign” and normal LV systolic function present.

**Additional file 7.** **Case 3 Movie 4.** Short axis cine bSSFP stack. Dilated RV with globally mildly reduced systolic function present. Mild LV dilation with normal systolic function and no regional wall motion abnormalities present.

**Additional file 8.** **Case 4 Movie 1**. Two chamber (**A**), three chamber (**B**), four chamber **C** cine bSSFP. Severely dilated LV with severely decreased systolic function, aortic valve stenosis, and mitral valve stenosis and regurgitation.

**Additional file 9.** **Case 5 Movie 1.** Two chamber (**A**), three chamber (**B**), and four chamber **C** cine bSSFP. There is moderately decreased left ventricular systolic function.

**Additional file 10.** **Case 6 Movie 1.** Three chamber **A** and short axis stack **B** bSSFP cine images. These images show a dilated LV, dyskinesis of inferolateral wall and decreased global systolic function.

**Additional file 11.** **Case 7 Movie 1.** Single shot bSSFP axial stack. No evidence of pulmonary consolidations and no pleural or pericardial effusions present.

**Additional file 12.** **Case 7 Movie 2.** Two chamber (**A**), three chamber (**B**), four chamber (**C**), and mid short axis **D** cine SSFP. Normal LV volumes, wall thickness, and ejection fraction without wall motion abnormalities.

**Additional file 13.** **Case 7 Movie 3. **Short axis stack LGE. Likely patchy mid-myocardial LGE affecting the mid anterior and mid to apical anteroseptal walls.

**Additional file 14.** **Case 8 Movie 1.** Contrast enhanced TTE four chamber and short axis views. TTE showed multiple wall motion abnormalities as well as the presence of LV masses.

**Additional file 15.** **Case 8 Movie 2.** Cine bSSFP two chamber (**A**), short axis (**B**), and three chamber views (**C**). The LV was severely dilated with severely reduced systolic function. The basal and mid inferior and inferolateral walls were thin, aneurysmal with a large mass present, and the entire apex was thin and akinetic with another mass present.

**Additional file 16.** **Case 8 Movie 3.** First pass perfusion four chamber **A** and short axis **B** views. There is no uptake of contrast in either mass.

**Additional file 17.** **Case 9 Movie 1.** Four chamber (**A**), mid short axis (**B**), and three (chamber) cine balanced bSSFP images. Low normal biventricular systolic function and marked pericardial thickening with a moderate left pleural effusion.

**Additional file 18.** **Case 9 Movie 2.** Real time four chamber **A** and mid short axis **B** cine. No evidence of interventricular dependence present.

## Data Availability

All data generated or analyzed during this study are included in this published article [and its additional files].

## Abbreviations

ACS: Acute coronary syndrome
ACE: Angiotensin converting enzyme
ARVC: Arrhythmogenic right ventricular cardiomyopathy
AS: Aortic stenosis
AVR: Aortic valve replacement
BiPAP: Bi-level positive airway pressure
BMI: Body mass index
bpm: Beats per minute
bSSFP: Balanced steady state free precession
BUN: Blood urea nitrogen
CABG: Coronary artery bypass grafting
CAD: Coronary artery disease
CMR: Cardiovascular magnetic resonance
COVID-19: Coronavirus disease 2019
CRP: C-reactive protein
CT: Computed tomography
DNI: Do not intubate
DNR: Do not resuscitate
DSG2: Desmoglein-2
ECG: Electrocardiogram
ECV: Extracellular volume
eGFR: Estimated glomerular filtration rate
EMB: Endomyocardial biopsy
FIDDLE: Flow independent dark blood delayed enhancement
HCM: Hypertrophic cardiomyopathy
HES: Hypereosinophilic syndromes
HF: Heart failure
ICD: Implantable cardioverter defibrillator
ICU: Intensive care unit
LA: Left atrium/left atrial
LAD: Left anterior descending coronary artery
LCx: Left circumflex coronary artery
LGE: Late gadolinium enhancement
LV: Left ventricle / left ventricular
LVEDVI: Left ventricular end-diastolic volume index
LVEF: Left ventricular ejection fraction
LVESVI: Left ventricular end-diastolic volume index
LVH: Left ventricular hypertrophy
MI: Myocardial infarction
MIS-A: Multisystem inflammatory syndrome in adults
NSTEMI: Non-ST-elevation myocardial infarction
NT-pro BNP: N-terminal prohormonte brain natriuretic peptide
PCI: Percutaneous coronary intervention
PCR: Polymerase chain reaction
PET: Positron emission tomography
PVCs: Premature ventricular contractions
RA: Right atrium/right atrial
RCA: Right coronary artery
RA Right atrium/right atrial RV: Right ventricle / right ventricular
RVEDVI: Right ventricular end-diastolic volume index
RVEF: Right ventricular ejection fraction
RVESVI: Right ventricular end-systolic volume index
SARS-CoV-2: Severe acute respiratory syndrome coronavirus 2
SCMR: Society for Cardiovascular Magnetic Resonance
SPECT: Single photon emission computed tomography
SSFP: Steady state free precession
STIR: Short tau inversion recovery
TAVR: Transcatheter aortic valve replacement
TIMI: Thrombolysis in myocardial infarction
TMVR: Transcatheter mitral valve replacement
TTE: Transthoracic echocardiogram
VT: Ventricular tachycardia
VTE: Venous thromboembolism
